# Profiling cancer-associated genetic alterations and molecular classification of cancer in Korean gastric cancer patients

**DOI:** 10.18632/oncotarget.19435

**Published:** 2017-07-22

**Authors:** Yoonjung Kim, Mee-Yon Cho, Juwon Kim, Sung Nam Kim, Seoung Chul Oh, Kyung-A Lee

**Affiliations:** ^1^ Department of Laboratory Medicine, Yonsei University College of Medicine, Seoul, Korea; ^2^ Department of Pathology, Yonsei University Wonju College of Medicine, Wonju, Korea; ^3^ Department of Laboratory Medicine, Yonsei University Wonju College of Medicine, Wonju, Korea; ^4^ Department of Pathology, Samkwang Medical Labotories, Seoul, Korea; ^5^ Department of Laboratory Medicine, Gangnam Severance Hospital, Seoul, Korea

**Keywords:** gastric cancer, molecular subtyping, germline mutation, somatic mutaiton, targeted sequencing

## Abstract

Recently, the Cancer Genome Atlas (TCGA) Research Network and Asian Cancer Research Group provided a new classification of gastric cancer (GC) to aid the development of biomarkers for targeted therapy and predict prognosis. We studied associations between genetically aberrant profiles of cancer-related genes, environmental factors, and histopathological features in 107 paired gastric tumor-non-tumor tissue GC samples. 6.5% of our GC cases were classified as the EBV subtype, 17.8% as the MSI subtype, 43.0% as the CIN subtype, and 32.7% as the GS subtype. The distribution of four GC subgroups based on the TCGA and our dataset were similar. The MSI subtype showed a hyper-mutated status and the best prognosis among molecular subtype. However, molecular classification based on the four GC subtypes showed no significant survival differences in terms of overall survival (*p*= 0.548) or relapse-free survival (RFS, *p*=0.518). The P619fs*43 in *ZBTB20* was limited to MSI group (n= 5/19, 26.3%), showing similar trends observed in TCGA dataset.

Genetic alterations of the RTK/RAS/MAPK and PI3K/AKT/mTOR pathways were detected in 34.6% of GC cases (37 individual cases). We also found two cases with likely pathogenic variants (NM_004360.4: c. 2494 G>A, *p.V832M*) in the *CDH1* gene.

Here, we classified molecular subtypes of GC according to the TCGA system and provide a critical starting point for the design of more appropriate clinical trials based on a comprehensive analysis of genetic alterations in Korean GC patients.

## INTRODUCTION

Gastric cancer (GC) is ranked fifth for cancer incidence and second for cancer deaths, and one in 36 men and 1 in 84 women develop stomach cancer before age 79 [1]. The histologic classification of gastric carcinoma has been based on the Lauren [2] and 2010 WHO classification systems, which recognize four histological subtypes [3]. Neither the Lauren nor the WHO system is particularly clinically useful, as their prognostic and predictive capabilities cannot adequately guide patient management. Thus, new classifications are needed for GC to provide insights into pathogenesis and the identification of new biomarkers and novel treatment targets [4]. Recently, advances in technology and high-throughput analysis have improved our understanding of the genetic basis of GC. To provide a roadmap for patient stratification and trials of targeted therapies, the Cancer Genome Atlas (TCGA) Research Network has characterized 295 primary gastric adenocarcinomas and proposed a new classification of four different tumor subtypes of *Epstein-Barr* virus (EBV)-positive, microsatellite instability (MSI), genomically stable (GS), and chromosomal instability (CIN) subtypes. [5]. The Asian Cancer Research Group (ACRG) also provided a new classification for GC, identifying four subtypes: MSI, MSS/EMT, MSS/TP53 (+), and MSS/TP53(–). One of the most important aspects of the ACRG classification is that it correlates the molecular subtypes with clinical prognosis [6].

*Helicobacter pylori* has been accepted as a causative organism in GC [7, 8], and EBV is also now regarded as a GC-causing infectious agent. The original type of EBV-infected GC makes up 5-10% of all GC cases [9, 10]. The vast majority of GC arise sporadically, and an inherited component contributes to <3% of gastric cancers [11, 12]. The GC is a heterogeneous disease characterized by epidemiologic and histopathologic differences across countries [7, 8, 13, 14]. Here, we investigated germline mutations in *CDH1, MSH2, MLH1, TP53, APC,* and *STK11,* which are associated with hereditary diffuse gastric cancer, hereditary nonpolyposis colon cancer, Li–Fraumeni syndrome, familial adenomatous polyposis, and Peutz–Jeghers syndrome, respectively [11, 12, 15–19]. Furthermore, we studied associations between the genetic aberrant profiles of cancer-related genes, environmental factors (*EBV* and *H. pylori*), and histopathological features in Korean GC patients. We also classified molecular subtypes of Korea GC.

## RESULTS

### Clinical and pathological findings of 107 Korean GC patients

The male to female ratio was 2:1 with median patient age of 70 years (range, 32-90). The intestinal type, diffuse type, and mixed type by Lauren classification accounted for 54.2%, 26.2%, and 19.6%, respectively. In addition, for GC classified according to the 2010 WHO classification, the tubular type (64.5%) was observed with the highest frequency, while the poorly cohesive type and mixed type accounted for 13.1% and 15.0%, respectively. More than half of the tumors were located in the antrum or antrum body, while about 7.5% were located in the cardia and gastroesophageal junction. Fifty-two cases (48.6%) were in stages III-IV, and 66 (61.7%) were *H.pylori*-positive. The clinicopathological findings of the 107 GC patients are summarized in Table 1.

**Table 1:** Clinicopathological characteristics of patients with gastric cancer (n=107)

Characteristics	Number
**Age (year)**	
Median (range)	70 (32 ∼ 90)
**Sex**	
Female	36 (33.6%)
Male	71 (66.4%)
**Lauren class**	
Diffuse	28 (26.2%)
Intestinal	58 (54.2%)
Mixed	21 (19.6%)
**WHO class**	
Mucinous	3 (2.8%)
Tubular	69 (64.5%)
Poorly cohesive	14 (13.1%)
Mixed (Tubular_Poorly cohesive)	16 (15.0%)
Uncommon histologic variants	5 (4.7%)
**pT stage**	
T1a/T1b	9 (8.4%)/15 (14.0%)
T2	11 (10.3%)
T3	38 (35.5%)
T4a/T4b	33 (30.8%)/1 (0.9%)
**pN stage**	
N0/N1/N2/N3	40 (37.4%)/16 (15.0%)/20 (18.7%)/31 (29.0%)
**M stage**	
M0/M1	102 (95.3%)/5 (4.7%)
**AJCC stage**	
Stages IA/IB	18 (16.8%)/11 (10.3%)
Stages IIA/IIB	13 (12.1%)/13 (12.1%)
Stages IIIA/IIIB/IIIC	17(15.9%)/11 (10.3%)/19 (17.8%)
Stage IV	5 (4.7%)
**Anatomical regions**	
GEJ_Cardia	8 (7.5%)
Fundus_Body	36 (34.3%)
Antrum	54 (50.5%)
Antrum_Body	5 (4.7%)
Pylorus	3 (2.8%)
Diffuse	1 (0.9%)
**Epstein-Barr virus infection**	
Negative	100 (93.5%)
Positive	7 (6.5%)
**Microsatellite instability(MSI)**	
MSS	88 (82.2%)
MSI-I	4 (3.7%)
MSI-H	15 (14.0%)
***H. pylori* infection**	
Negative	41 (38.3%)
Positive	66 (61.7%)

Abbreviations: GEJ, gastroesophageal junction; *H. pylori, Helicobacter pylori;* pT stage, pathological assessment of the primary tumor; pN stage, pathological assessment of the regional lymph nodes.

### Germline variation analysis of hereditary cancer-predisposing syndrome

A total of 30 germline variants were observed in *TP53, STK11, ALK, APC, MSH2, MLH1,* and *CDH1* (Table 2). Among these, 16 variants, 12 variants, and 2 variants were classified as 'Benign or Likely Benign,' 'VUS,' and 'Likely pathogenic,' respectively. Two cases had a likely pathogenic variant (*p.V832M*) in *CDH1* gene.

**Table 2:** Germline SNPs in *TP53, STK11, ALK, APC, MSH2, MLH1*, and *CDH1* genes of 107 Korean gastric cancer patients

Sample	Chromosome	Start	End	Ref	Alter	Gene	Transcript ID	Amino acid change	Total depth	VAF(%)(1)^a^	VAF (%)(2)^b^	Clinical significance
YMC 54	2	29449820	29449820	G	A	ALK	NM_004304.4	p.T1012M	637	48.51%	60.36%	Benign
YMC 7	2	29449820	29449820	G	A	ALK	NM_004304.4	p.T1012M	208	55.77%	43.83%	Benign
YMC 70	2	29449820	29449820	G	A	ALK	NM_004304.4	p.T1012M	755	52.58%	57.85%	Benign
SKW 18	2	29519923	29519923	G	A	ALK	NM_004304.4	p.L550F	155	38.06%	55.39%	VUS
YMC 13	5	112177778	112177778	A	C	APC	NM_001127511.2	p.K2145Q	151	52.32%	33.74%	Benign
YMC 24	5	112178865	112178865	G	A	APC	NM_001127511.2	p.R2507H	1142	47.90%	49.52%	VUS
SKW 38	5	112176548	112176548	G	C	APC	NM_001127511.2	p.A1735P	628	45.86%	47.30%	VUS
SKW 21	5	112173895	112173895	A	C	APC	NM_001127511.2	p.E850D	147	55.78%	50.93%	Benign
YMC 29	16	68867247	68867247	G	A	CDH1	NM_004360.4	p.V832M	1337	52.21%	49.75%	Likely pathogenic
YMC 37	16	68867247	68867247	G	A	CDH1	NM_004360.4	p.V832M	1498	50.73%	46.41%	Likely pathogenic
SKW 16	16	68856080	68856080	C	G	CDH1	NM_004360.4	p.L630V	928	43.74%	40.61%	Benign
SKW 15	16	68856080	68856080	C	G	CDH1	NM_004360.4	p.L630V	827	49.33%	71.46%	Benign
YMC 53	3	37053562	37053562	C	T	MLH1	NM_000249.3	p.R217C	1397	46.96%	50.40%	VUS
YMC 55	3	37042521	37042521	T	G	MLH1	NM_000249.3	p.S95A	233	39.06%	30.52%	VUS
YMC 6	3	37067240	37067240	T	A	MLH1	NM_000249.3	p.V384D	578	48.79%	44.85%	Benign
YMC 70	3	37067240	37067240	T	A	MLH1	NM_000249.3	p.V384D	547	51.55%	47.99%	Benign
YMC 14	3	37053562	37053562	C	T	MLH1	NM_000249.3	p.R217C	1409	45.71%	51.65%	VUS
YMC 22	3	37067240	37067240	T	A	MLH1	NM_000249.3	p.V384D	862	99.54%	100%	Benign
YMC 3	3	37090506	37090506	C	A	MLH1	NM_000249.3	p.Q701K	369	51.49%	41.29%	Benign
YMC 4	3	37089022	37089022	C	G	MLH1	NM_000249.3	p.L582V	364	48.63%	57.47%	VUS
YMC 37	3	37053562	37053562	C	T	MLH1	NM_000249.3	p.R217C	1315	49.20%	49.76%	VUS
YMC 48	3	37067240	37067240	T	A	MLH1	NM_000249.3	p.V384D	899	43.38%	44.61%	Benign
SKW 31	3	37053562	37053562	C	T	MLH1	NM_000249.3	p.R217C	294	55.78%	39.71%	VUS
YMC 66	2	47656972	47656972	C	T	MSH2	NM_000251.2	p.L390F	197	56.85%	52.78%	Benign
YMC 15	2	47656972	47656972	C	T	MSH2	NM_000251.2	p.L390F	1281	46.68%	79.62%	Benign
YMC 28	2	47630344	47630344	C	A	MSH2	NM_000251.2	p.P5Q	231	53.25%	86.79%	VUS
YMC 5	2	47637371	47637371	A	G	MSH2	NM_000251.2	p.I169V	711	51.34%	45.12%	Likely benign
YMC 48	2	47656972	47656972	C	T	MSH2	NM_000251.2	p.L390F	692	51.30%	53.31%	Benign
SKW 41	2	47703564	47703564	G	A	MSH2	NM_000251.2	p.M688I	993	52.57%	50.83%	VUS
SKW 40	17	7578209	7578209	G	A	TP53	NM_000546.5	p.H214Y	168	39.29%	59.58%	VUS

^a^, tumor tissue; ^b^, matched non-tumor tissue.

Abbreviation: Ref, reference allele; Alter, Altered allele; VAF, Variant allele frequency; VUS, a variant of unknown significance.

### Molecular profile of gastric cancer with a 43 gene cancer panel

To identify driver genes [20] causally linked to tumorigenesis in Korea GCs, variants were extracted from targeted sequencing with a 43 gene cancer panel applied to 107 tumor and matched non-tumor tissues. After mutation calling and stringent filtrations, we identified 317 single-nucleotide variants (SNVs) on coding sequences that included missense variations (n=164, 51.7%), trunc (nonsense and frameshift) variations (n= 110, 34.7%), in-frame variations (n= 42, 13.2%), and splicing site variations (n=1, 0.3%) (Supplementary Table 2). Somatic variants detected in each gene for each sample were summarized in Supplementary Table 3. Among the 43 genes, we discovered 39 genes that were mutated in one or more individual samples. Among the 107 samples, *TP53* (38.3%), *ARID1A* (36.4%), *CR1* (15.0%), *APC* (11.2%), *BCOR* (11.2%), *CDH1* (10.3%), *CIC* (9.3%), *PIK3CA* (9.3%), *ERBB3* (8.4%), *RHOA* (8.4%), *ERBB2* (7.5%), CCND1 (6.5%), *FBXW7* (6.5%), *ALK* (5.6%), *KRAS* (5.6%), and *MTOR* (5.6%) were identified (Supplementary Table 3). Somatic variants were detected in relatively low frequencies (less than 5%) on the *CTNNB1, EGFR, HLA-B, MSH2, PGM5, ZBTB20, IRF2, KDR, LARP4B, MVK, BRAF, PTEN, ACVR1B, CBWD1, FGFR2, JAK2, MEDAG, MLH1, SMAD4, STK11, CD274, MDM2,* and *MYC* genes in this study. We did not detect any somatic variants on the *C16orf74, CCNE1, PDCD1LG2,* or *MET* genes (Supplementary Table 3).

**Figure 1:**
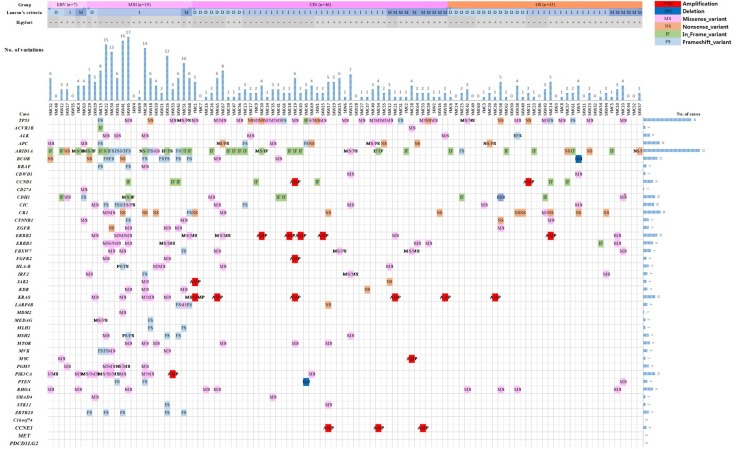
Summary of somatic mutations in 107 gastric cancer samples according to molecular subtype

Among 317 somatic variants, 110 (34.7%) were recurrently detected in this study (Table 3). According to the results of somatic variants, *p.Gln1334del/dup* or *p.Asp1850ThrfsTer33* in *ARID1A, p.Arg2194Ter* in *CR1, p.Glu280del* in *CCND1*, *p.Leu15del* in *CDH1, p.Arg678Gln* in *ERBB2, p.Gly13Asp* in *KRAS, p.His1047Arg* in *PIK3CA,* and *p.Pro619LeufsTer43* in *ZBTB20* were recurrently detected in more than four individual cases (Table 3). Twenty-two copy number variation (CNV)s were identified in the *BCOR* (n=1), *CCND1* (n=2), *CCNE1* (n=3), *ERBB2* (n=5), *FGFR2* (n=1), *KRAS* (n=6), *MYC* (n=1), *PIK3CA* (n=1), *PTEN* (n=1), and *JAK2* (n=1) (Figure 1).

**Table 3:** The location-specific recurrence of somatic variants in the 43 cancer panel genes

Gene	Variants^a^	Mutated samples	% of mutated samples	COSMIC counts	COSMIC ID
*APC*	*p.Glu1464ValfsTer8*	2	1.90%	45	COSM1432412; COSM19694; COSM41622; COSM41622; COSM41622; COSM5030795
*ARID1A*	*p.Asp1850ThrfsTer33*	5	4.70%	27	COSM1341426; COSM133001; COSM1666860
*ARID1A*	*p.Gln1334del/dup*	23	21.50%	23	COSM1341408; COSM298325; COSM1578346; COSM133030; COSM51218; COSM1238047
*ARID1A*	*p.Gly87del*	2	1.90%		(-)
*BCOR*	*p.Gln1174ThrfsTer8*	3	2.80%	5	COSM1683572; COSM3732385
*BRAF*	*p.Pro403LeufsTer8*	2	1.90%	7	COSM1448632; COSM5347158; COSM5347157
*CCND1*	*p.Glu280del*	7	6.50%	3	COSM931394
*CDH1*	*p.Leu15del*	5	4.70%		(-)
*CR1*	*p.Arg2194Ter*	10	9.30%	20	COSM301989
*ERBB2*	*p.Arg678Gln*	4	3.70%	23	COSM978678; COSM436498
*ERBB2*	*p.Ser310Phe*	2	1.90%	51	COSM48358; COSM1666868
*ERBB2*	*p.Val842Ile*	2	1.90%	26	COSM14065; COSM1666633
*ERBB3*	*p.Val104Met*	2	1.90%	21	COSM20710
*FBXW7*	*p.Arg385His*	3	2.80%	41	COSM117308
*HLA-B*	*p.Glu69Val*	2	1.90%	3	COSM4598273
*KRAS*	*p.Gly13Asp*	5	4.70%	5039	COSM532
*LARP4B*	*p.Thr163HisfsTer47*	2	1.90%	27	COSM1638669; COSM4968611
*MVK*	*p.Ala141ArgfsTer18*	2	1.90%	17	COSM1241457
*PIK3CA*	*p.His1047Arg*	4	3.70%	2115	COSM775
*PGM5*	*p.Ile98Val*	3	2.80%	47	COSM1109610
*RHOA*	*p. Arg5Gln/Trp*	3	2.80%	23	COSM446704; COSM190569; COSM4770224; COSM4770223
*RHOA*	*p.Thr37Ala/Ile*	2	1.90%	3	COSM5064959; COSM1223700
*RHOA*	*p.Tyr42Cys*	2	1.90%	22	COSM2849892; COSM4770225
*TP53*	*p.Arg248Trp*	2	1.90%	56	COSM144150
*TP53*	*p.Arg342Ter*	2	1.90%	151	COSM11073
*TP53*	*p.Cys275Tyr*	2	1.90%	62	COSM10893
*TP53*	*p.Val173Leu*	2	1.90%	65	COSM43559
*ZBTB20*	*p.Pro619LeufsTer43*	5	4.70%	26	COSM267785

^a^Recurrent mutations observed in at least two samples.

**Figure 2:**
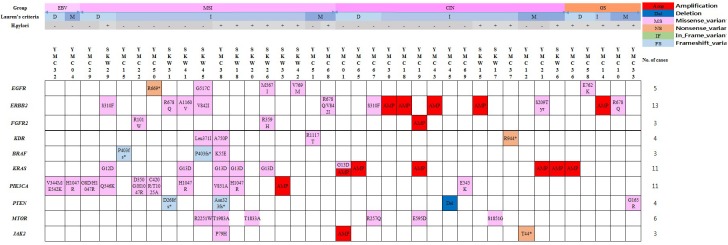
Therapeutic implications of somatic genomic alterations in 107 clinical gastric cancer cases

Genetic alterations of the RTK/RAS/MAPK or/and PI3K/AKT/mTOR pathways were detected in 34.6% of GC cases (37 individual cases) (Figure 2). Receptor tyrosine kinase (RTK) genomic alterations including *ERBB2* (n=13), *EGFR* (n=5), *FGFR2* (n=3) and *KDR* (n=4) were detected in 20.5% of GC cases (n=22). Thirteen samples (12.2% of GC) harbored ERBB2 alterations, 8 contained somatic base substitutions and 5 harbored amplifications, with these events being mutually exclusive. Genetic alterations of *PTEN* (n=4), *PIK3CA* (n=11), *K*RAS (n=11), *BRAF* (n=3) and *MTOR* (n=6) were detected in 25.2% of GC cases (n=27). Ten cases (9.4%) harbored mutated *PIK3CA* and *KRAS* G13D co-existed in 4 cases (Figure 2).

### Molecular subtype classification and clinical phenotype

We classified molecular subtypes using genomic data according to subtypes derived by TCGA and correlated clinical covariates of 107 GC patients with those molecular subtypes (Table 4). The EBV subtype (6.5% of GC) was significantly enriched in EBV burden and characterized as uncommon histological subtype (Table 5). In the EBV subtype, no samples with a *TP53* mutation were detected, but mutations of *ARID1A* (4 cases, 57.1%), *CDH1* (3 cases, 42.9%), *PIK3CA* (2 cases, 28.6%) and *RHOA* (2 cases, 28.6%) were present with a relatively high frequency. Genetic alterations of the *JAK2* and *PDCD1LG2* genes were not detected in the EBV subgroup. Only one case harbored the mutant *CD274* (Figure 1 & Table 4).

**Table 4:** Somatic mutations in each subtype

Somatic mutations	EBV (N=7)		MSI (N=19)		CIN (N=46)		GS (N=35)		*P* value
	Cases	(%)	Cases	(%)	Cases	(%)	Cases	(%)	
TP53	0	0.0%	5	26.3%	26	56.5%	10	28.6%	0.003^*a*^
ACVR1B	0	0.0%	1	5.3%	1	2.2%	0	0.0%	0.534
ALK	0	0.0%	3	15.8%	2	4.3%	1	2.9%	0.232
APC	2	28.6%	1	5.3%	8	17.4%	1	2.9%	0.050
ARID1A	4	57.1%	14	73.7%	12	26.1%	9	25.7%	0.000^*a*^
BCOR	1	14.3%	9	47.4%	2	4.3%	0	0.0%	0.016^*a*^
BRAF	0	0.0%	3	15.8%	0	0.0%	0	0.0%	0.003^*a*^
CBWD1	0	0.0%	0	0.0%	1	2.2%	1	2.9%	1.000
CCND1	0	0.0%	3	15.8%	2	4.3%	2	5.7%	0.323
CD274	1	14.3%	0	0.0%	0	0.0%	0	0.0%	0.065
CDH1	3	42.9%	1	5.3%	3	6.5%	4	11.4%	0.052
CIC	0	0.0%	5	26.3%	3	6.5%	2	5.7%	0.082
CR1	0	0.0%	5	26.3%	2	4.3%	8	22.9%	0.220
CTNNB1	1	14.3%	2	10.5%	0	0.0%	2	5.7%	0.054
EGFR	0	0.0%	4	21.1%	0	0.0%	1	2.9%	0.005^*a*^
ERBB2	0	0.0%	5	26.3%	2	4.3%	1	2.9%	0.028^*a*^
ERBB3	0	0.0%	5	26.3%	2	4.3%	2	5.7%	0.049^*a*^
FBXW7	0	0.0%	3	15.8%	3	6.5%	1	2.9%	0.301
FGFR2	0	0.0%	2	10.5%	0	0.0%	0	0.0%	0.057
HLA-B	0	0.0%	3	15.8%	2	4.3%	0	0.0%	0.088
IRF2	0	0.0%	2	10.5%	1	2.2%	1	2.9%	0.429
JAK2	0	0.0%	1	5.3%	1	2.2%	0	0.0%	0.534
KDR	0	0.0%	3	15.8%	1	2.2%	0	0.0%	0.046^*a*^
KRAS	0	0.0%	5	26.3%	1	2.2%	0	0.0%	0.002^*a*^
LARP4B	0	0.0%	3	15.8%	1	2.2%	0	0.0%	0.046^*a*^
MDM2	0	0.0%	1	5.3%	0	0.0%	0	0.0%	0.243
MEDAG	0	0.0%	2	10.5%	0	0.0%	0	0.0%	0.057
MLH1	0	0.0%	2	10.5%	0	0.0%	0	0.0%	0.057
MSH2	0	0.0%	4	21.1%	1	2.2%	0	0.0%	0.009^*a*^
MTOR	0	0.0%	3	15.8%	3	6.5%	0	0.0%	0.111
MVK	0	0.0%	4	21.1%	0	0.0%	0	0.0%	0.002^*a*^
MYC	1	14.3%	0	0.0%	0	0.0%	0	0.0%	0.065
PGM5	1	14.3%	4	21.1%	0	0.0%	0	0.0%	0.001^*a*^
PIK3CA	2	28.6%	7	36.8%	1	2.2%	0	0.0%	0.000^*a*^
PTEN	0	0.0%	2	10.5%	0	0.0%	1	2.9%	0.096
RHOA	2	28.6%	1	5.3%	2	4.3%	4	11.4%	0.139
SMAD4	0	0.0%	1	5.3%	1	2.2%	0	0.0%	0.534
STK11	0	0.0%	1	5.3%	1	2.2%	0	0.0%	0.534
ZBTB20	0	0.0%	5	26.3%	0	0.0%	0	0.0%	0.000^*a*^
Mutation rate	2.6		6.6		1.8		1.5		

^*a*^*p* value less than 0.05.

Abbreviations: CIN, chromosomal instability, EBV, Epstein-Barr virus; GS, genomically stable; MSI, microsatellite instability. Fisher`s exact test was performed to evaluate differences in the respective proportions of somatic mutations between subgroups.

**Table 5:** Patient characteristics according to molecular subtype

	EBV (n=7)		MSI (n=19)		CIN (n=46)		GS (n=35)		*P* value
	Cases	(%)	Cases	(%)	Cases	(%)	Cases	(%)	
**Age**									
≤50	0	0.0%	1	5.3%	3	6.5%	6	17.1%	0.580
51 ∼60	2	28.6%	1	5.3%	9	19.6%	6	17.1%	
61 ∼70	1	14.3%	3	15.8%	13	28.3%	8	22.9%	
71 ∼ 80	3	42.9%	12	63.2%	16	34.8%	13	37.1%	
>80	1	14.3%	2	10.5%	5	10.9%	2	5.7%	
**Sex**									
Female	1	14.3%	8	42.1%	16	34.8%	11	31.4%	0.660
Male	6	85.7%	11	57.9%	30	65.2%	24	68.6%	
**Lauren Class**									
Diffuse	3	42.9%	2	10.5%	9	19.6%	14	40.0%	0.069
Intestinal	2	28.6%	15	78.9%	26	56.5%	15	42.9%	
Mixed	2	28.6%	2	10.5%	11	23.9%	6	17.1%	
**WHO Class**									
Tubular	2	28.6%	17	89.5%	32	69.6%	18	51.4%	0.000
Mucinous	0	0.0%	0	0.0%	2	4.3%	1	2.9%	
Poorly cohesive	0	0.0%	0	0.0%	5	10.9%	9	25.7%	
Mixed	1	14.3%	1	5.3%	7	15.2%	7	20.0%	
Uncommon	4	57.1%	1	5.3%	0	0.0%	0	0.0%	
**pT stages**									
T1a/ T1b	2	28.6%	2	10.5%	10	21.7%	10	28.6%	0.778
T2	1	14.3%	3	15.8%	4	8.7%	3	8.6%	
T3	1	14.3%	9	47.4%	17	37.0%	11	31.4%	
T4a/ T4b	3	42.9%	5	26.3%	15	32.6%	11	31.4%	
**p N stages**									
N0	3	42.9%	10	52.6%	16	34.8%	11	31.4%	0.802
N1	1	14.3%	3	15.8%	7	15.2%	5	14.3%	
N2	1	14.3%	4	21.1%	9	19.6%	6	17.1%	
N3	2	28.6%	2	10.5%	14	30.4%	13	37.1%	
**M stages**									
M0	6	85.7%	19	100.0%	43	93.5%	34	97.1%	0.351
M1	1	14.3%	0	0.0%	3	6.5%	1	2.9%	
**AJCC Stages**									
Stages IA/IB	3	42.9%	4	21.1%	11	23.9%	11	31.4%	0.605
Stages IIA/IIB	1	14.3%	9	47.4%	13	28.3%	3	8.6%	
Stages IIIA/IIIB/IIIC	2	28.6%	6	31.6%	19	41.3%	20	57.1%	
Stage IV	1	14.3%	0	0.0%	3	6.5%	1	2.9%	
**Anatomical regions**									
Antrum	1	14.3%	15	78.9%	25	54.3%	13	37.1%	0.004
Antrum_Body	1	14.3%	0	0.0%	2	4.3%	2	5.7%	
Fundus_Body	4	57.1%	4	21.1%	10	21.7%	18	51.4%	
GEJ_Cardia	1	14.3%	0	0.0%	7	15.2%	0	0.0%	
Diffuse	0	0.0%	0	0.0%	0	0.0%	1	2.9%	
Pylorus	0	0.0%	0	0.0%	2	4.3%	1	2.9%	
**Lymphatic invasion**									
Positive	4	57.1%	17	89.5%	34	73.9%	21	60.0%	0.125
Negative	3	42.9%	2	10.5%	12	26.1%	13	37.1%	
Missing	0	0.0%	0	0.0%	0	0.0%	1	2.9%	
**Venous invasion**									
Positive	2	28.6%	1	5.3%	7	15.2%	5	14.3%	0.416
Negative	5	71.4%	18	94.7%	39	84.8%	29	82.9%	
Missing	0	0.0%	0	0.0%	0	0.0%	1	2.9%	
**Perineural invasion**									
Positive	4	57.1%	6	31.6%	25	54.3%	17	48.6%	0.391
Negative	3	42.9%	13	68.4%	21	45.7%	17	48.6%	
Missing	0	0.0%	0	0.0%	0	0.0%	1	2.9%	
***H. pylori* infection**									
Negative	3	42.9%	8	42.1%	21	45.7%	9	25.7%	0.290
Positive	4	57.1%	11	57.9%	25	54.3%	26	74.3%	

Abbreviations: CIN, chromosomal instability, EBV, Epstein-Barr virus; GS, genomically stable; gastric cancer, GC; GEJ, gastroesophageal junction; *H. pylori, Helicobacter pylori;* MSI, microsatellite instability; Mixed, mixed type with tubular and poorly cohesive; pT stage, pathological assessment of the primary tumor; pN stage, pathological assessment of the regional lymph nodes; Uncommon, uncommon histologic variants.

The MSI subtype (17.8% of GC) showed instability in one more locus in the MSI assay. The MSI subtype presented with an elevated mutation rate (6.6 per case) and was characterized by alterations of genes involved in mismatch repair. Almost all cases with mutations in *MLH1* (n=2/2) and *MSH2* (n=4/5) were included in the MSI subtype. Mutations of *ARID1A* (73.7%), *BCOR* (47.4%), *BRAF* (15.8%), *EGFR* (21.1%), *ERBB2* (26.3%), *ERBB3* (26.3%), *KDR* (15.8%), *KRAS* (26.3%), *LARP4B* (15.8%), *MSH2* (21.1%), *MVK (21.1%), PGM5* (21.1%), *PIK3CA* (36.8%) and *ZBTB20* (26.3%) were observed with statistical significance. Interestingly, we observed that mutations of *ZBTB20* were limited to the MSI group (Table 4)*.*

The CIN subtype (43.0% of GC) was also characterized by a relatively low-somatic mutation rate (1.8 per case) and a high frequency of *TP53* mutations. In the CIN subtype, we observed amplifications of *CCND1, CCNE1, ERBB2, FGFR2*, *KRAS, MYC, PIK3CA* and *JAK2,* and a deletion of *PTEN* among the 43 genes included in the Next-Generation Sequencing (NGS) panel. Ten cases (21.7% in the CIN subtype) harbored CNVs of genes belonging to the RTK/RAS/MAPK and PI3K/PTEN/AKT pathways (Figure 2). CNV detection using a targeted NGS panel of 43 genes represented 30.4 % of the CIN subtype (n=14/46) (Figure 1).

The GS subtype (32.7 % of GC) was characterized by a lack of EBV infection, MSI and somatic CNAs. The GS subtype showed the lowest mutation rate (1.5 per case) (Table 4).

### Prognosis analysis in 107 gastric cancer patients

**Figure 3:**
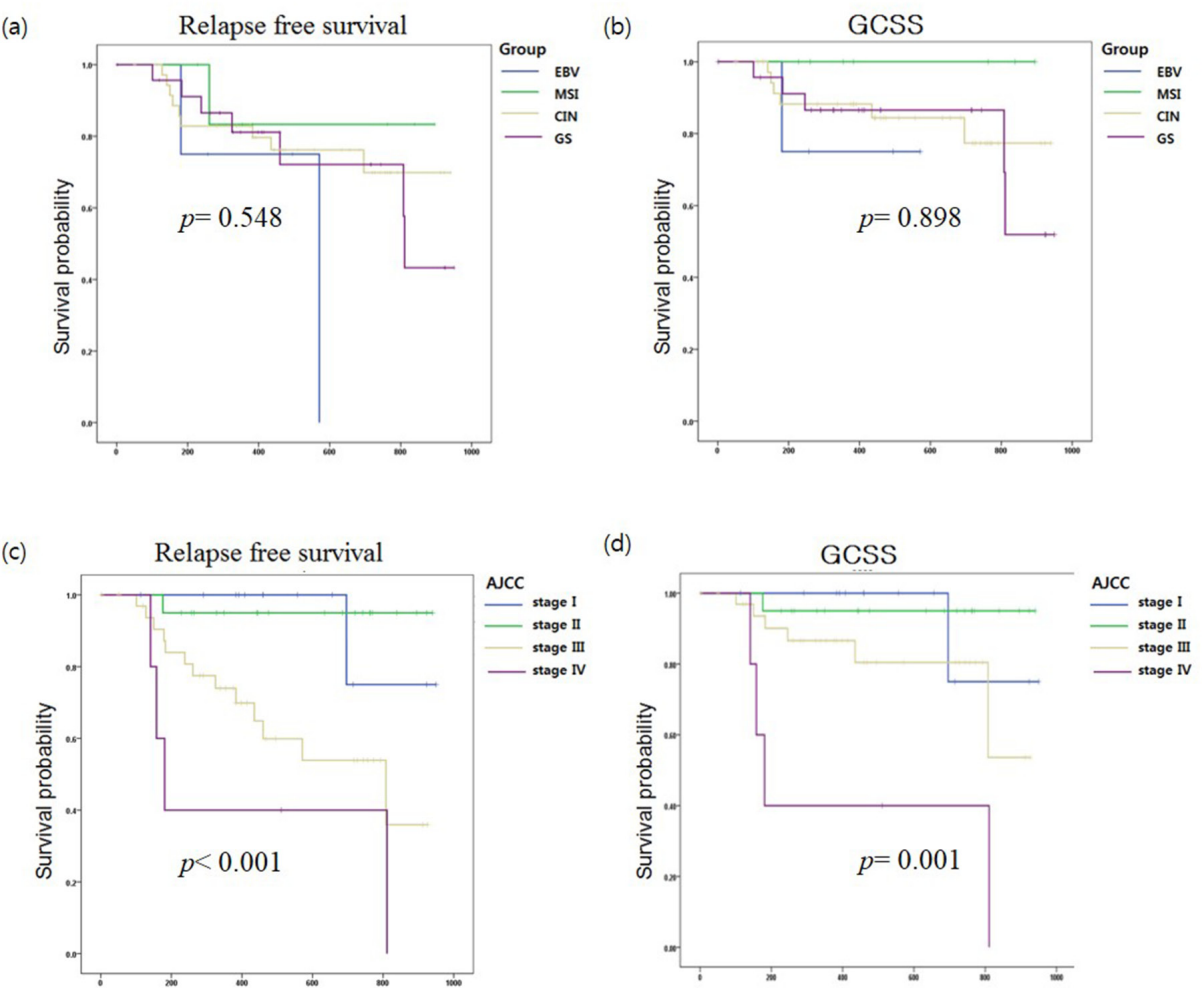
Kaplan-Meier **(a)** relapse-free survival (RFS) and **(b)** gastric cancer-specific survival (GCSS) curves were stratified by molecular subtypes of gastric cancer (EBV, MSI, CIN, and GS). Kaplan-Meier **(c)** RFS and **(d)** GCSS curves were analyzed by AJCC stage.

Among the 107 GC patients, the date of last follow-up (months), loco-regional recurrence, distant metastasis, and cause of death were obtained from 72 patients. The median follow up period was 459.5 days, and there were 19 (26.4%) and 12 (16.7%) cases of gastric cancer relapse and gastric cancer-related death, respectively. We conducted a survival analysis but did not observe a substantial difference in overall survival (*p*= 0.898) or relapse-free survival (RFS, *p*=0.548) among the four GC subtypes (Figure 3 (a) & (b)). The classification based on AJCC stages showed significant differences in overall survival (*p*= 0.001) or RFS (*p* <0.001) (Figures 3 (c) & (d)). Multivariate analysis showed mutant *MTOR* gene (hazard ratio [HR]:9.26, *p*= 0.002, 95% CI: 2.22 – 38.58), AJCC stages III (HR: 1.9, *p*= 0.013, 95% CI: 1.14 - 3.16), AJCC stages IV (HR: 2.15, *p*= 0.000, 95% CI: 1.40 - 3.30) and mutant *CCND1* gene (HR: 5.71, *p*= 0.028, 95% CI: 1.21 – 27.0) were associated with the relapse of GC.

## DISCUSSION

We analyzed germline mutations with paired non-tumor and GC tissue samples in 107 Korean patients. Two cases harbored a likely pathogenic variant (NM_004360.4: c. 2494 G>A, *p.V832M*) in the *CDH1* gene. A V832M mutation has been identified in a hereditary diffuse gastric cancer (HDGC) in a Japanese family. The probands were diagnosed at the age of 56 [21]. This mutation were functionally characterized as a pathogenic mutation [22] and it was also detected in familial lobular breast cancer patients with the wild type *BRCA1/2* gene [23]. Two cases with V832M were diagnosed at age 66 and 75, in this study, respectively. Both cases were advanced GC in stage IIB at diagnosis and the family history of GC was not known.

According to the results of somatic variants, the Q1334del/dup (n=23/52) in *ARID1A* and L15del (n=6/13) in *CDH1* were detected at a frequency of 5∼33% of altered alleles in tumor tissue (Table 3 & Supplementary 2). The in-frame indel (Q1334del/dup), which increases the amount of the ARID1A protein in the nucleus and restores its tumor suppressor functions, has also been reported in GC samples [24]. This single nucleotide polymorphism (SNP) were also occasionally reported in COSMIC database (COSMIC v78) and pancreatic cancers [25]. A three-nucleotide deletion c.44_46del TGC (L15del) in exon 1 of *CDH1,* which is in the signal peptide region of the E-cadherin protein, was also identified in Chinese GC patients, whereas it was not detected in 240 controls [26] and endometrial carcinomas [27]. RHOA belongs to the Rho family, which functions in the regulation of the actin cytoskeleton, and functional evidence indicates that mutant *RHOA* works in a gain-of-function manner in this gene [28]. An *RHOA* mutation was observed in 8.4% of GC cases (n=9/107), with mutations in the Arg5, Gly17, Thr37, Tyr42 and Glu64 residues (Table 3 & Supplementary 2). Among these mutations, the Arg5, Gly17, and Tyr42 residues are recurrently detected in GC [28, 29].

EBV-infected GC constitutes 5-10% of all GC cases [9, 10] and the Cancer Genome Atlas project demonstrated that EBV-infected GC is one of four molecular subtypes [28]. We also demonstrated that EBV-infected GC grouped as a molecular subtype. As in the EBV-subtype, *ARID1A* mutations (4 cases, 57.1%) were prevalent, and no samples with a *TP53* were detected. The frequency of *ARID1A* and *TP53* mutations were similar to the TCGA data [28]. Inhibitors of the PI3K/AKT/mTOR pathway, JAK2 pathway and *PD-1/PD-L1, PD-L2* pathway are considered as potentially applicable targeted therapies in EBV-infected GC [4, 30]. However, only 28.6 % of EBV-infected GC harbored *PIK3CA* mutations (n=2/7), and drug related amplifications of *JAK2, CD274*, *PDCD1LG2* and ERBB2 were not detected in the EBV subtype (Figure 2). To provide applicable therapeutic options, the genetic alterations of *PIK3CA AK2, CD274*, *PDCD1LG2* and *ERBB2* should be further validated with large-scale EBV-infected GC.

We observed that the MSI subtype was associated with hyper-mutations in genes and was characterized by a more favorable prognosis than other molecular subtypes. Both the TCGA and ACRG classifications also characterized the MSI subtype by the high mutation frequency and best prognosis [6, 28]. For intestinal type GC, patients with a good prognosis were characterized by a high mutation rate and microsatellite instability. Further, mutations of *PIK3CA* (29.4%) and *KRAS* (26.5%) were represented in good prognosis subgroup [31]. In our study, mutations of *KRAS* (26.3%) and *PIK3CA* (36.8%) were present with statistical significance in the MSI subtype, and *KRAS* G13D (4 cases) and *PIK3CA* H1047R mutations (3 cases) were frequently observed (Table 2). In addition, *PIK3CA* H1047R mutations were also frequently detected in the MSI subtype in a previous study [6]. The genetic alteration of *ZBTB20* (P619fs*43, n=5) was limited to the MSI group. This SNP (P619fs*43; rs758277701; COSM267785) also was limited to the MSI group and similar trend was observed (20% of MSI) in TCGA data [28]. The clinical significance of this variation should be evaluated through further studies.

The distribution of four GC subgroups based on the TCGA [28] and our data were similar (EBV, 8.8% vs. 6.5%; MSI, 21.7% vs. 17.8%; GS, 19.7% vs. 32.7 %; CIN, 49.8 % vs. 43.0 %). Furthermore, the proportion of EBV (6.4% vs. 6.5%), MSI (9.2% vs. 17.8%) and CIN subtype (51 % vs. 43.0 %) in Korean GC were also similar to our data. [32, 33].

Genetic alterations of the RTK/RAS/MAPK and PI3K/AKT/mTOR pathways were detected in 34.6% of GC cases (n=37) (Figure 2). Thirteen samples (12.2% of GC) harbored *ERBB2* alterations, 8 contained somatic base substitutions and 5 harbored amplifications, with these events being mutually exclusive. S310F (two cases) and V842I substitutions (two cases) in *ERBB2* were recurrently detected in this study and have been functionally characterized as activating and sensitive to lapatinib in ERBB2-negative breast cancers. The functions of *ERBB2* R678Q, which was also recurrently detected in this study, related to anti-ERBB2 (HER2)-targeted therapy has not been tested [34]. Ten cases (9.4%) harbored mutated *PIK3CA*, and *KRAS* G13D co-existed in 4 cases (Figure 2). Effects of the co-existence of genetic alterations of *PIK3CA* and *KRAS* on response to therapy are yet to be evaluated [4]. Dual PI3K and STAT3 blockade using NVP-BKM120 and AG490 (STAT3 inhibitor) showed a synergistic effect in GC cells harboring mutated *KRAS* by inducing apoptosis [35].

These biomarkers may facilitate enrollment of GC patients into clinical trials evaluating targeted therapies and provide the basis for developing solid therapeutic approaches in Korean GC patients [36–38].

Molecular classification based on four GC subtypes showed no significant survival differences in overall survival (*p*= 0.898) or RFS (*p*=0.548) in this dataset. And, ACRG classification-based subtypes also showed no significant association with survival in Korean GC [33]. Therefore, we thought that predicting prognosis for Korea GC patients might be performed more simply and effectively using AJCC stage [39] rather than molecular classification.

We classified molecular subtypes of gastric cancer according to the TCGA system using a targeted NGS panel of 43 genes, EBV, MSI, *H. pylori* and SNP array. The 43 gene cancer panel consisted of significantly mutated genes from the TCGA and ACRG cohort [6, 28, 40], genes associated with new targeted therapy of GC (*EGFR, ERBB2, FGFR2,* and *KDR*) and hereditary cancer syndromes (*CDH1, MSH2, MLH1, STK11,* and *TP53*) [12]. We demonstrated 1) the distribution of GC subtypes according to TCGA molecular group, 2) heritable genetic alterations, 3) environmental factors (*EBV* and *H. pylori*), 4) somatic genetic aberrant profiles including driver mutations and drug-targeted genetic alterations, and 5) histopathological features in Korean GC patients.

## MATERIALS AND METHODS

### Subject selection

We obtained a total of 107 gastric tumors and matched non-tumor tissue samples from Yonsei University Wonju Medical Center Biobank (n=138, 69 paired samples) and Samkwang Medical Laboratory Biobank (n= 76, 38 paired samples). Tumor samples were obtained from patients who had not received prior chemotherapy or radiotherapy. The gastric cancer tissues consisted of 69 fresh-frozen (FF) paired tumor and non-tumor tissue samples and 38 formalin-fixed paraffin-embedded (FFPE) paired tumor and non-tumor tissue samples. Clinical data, including age, sex, clinical follow-up data, and pathologic reports, were provided from the tissue source institutions. The histologic classification of gastric carcinoma has previously been based on Lauren’s criteria [2] and the 2010 WHO classification system [3]. Tumor TNM stage assignment was evaluated for consistency with the 7th Edition of the TNM classification by the American Joint Committee on Cancer (AJCC) [39]. Pathologic findings were reviewed by experienced gastrointestinal pathologists (S.N.K. and M.C.). The study was approved by the Institutional Review Boards of Samkwang Medical Laboratories and Yonsei University Wonju College of Medicine.

### DNA preparation

DNA was extracted from FFPE tumor and adjacent non-tumor gastric tissues using a QIAamp DNA extraction kit (Qiagen, Hilden, Germany) according to the manufacturer’s protocol. H&E-stained sections from FFPE blocks were reviewed by a board-certified pathologist, and representative sections with tumor content or benign tissue were identified. A G-DEX genomic DNA extraction kit (Intron Biotechnology, Korea) was used for FF tumor and matched non-tumor FF tissues according to the manufacturer’s protocol. The quality and concentration of genomic DNA (gDNA) was evaluated by Nanodrop (ND-1000; Thermo Scientific, DE, USA) and the Agilent 2200 Tape Station system (Agilent Technologies, CA, USA) with Genomic DNA Screen Tape according to the manufacturer’s instructions. The DNA Integrity Number (DIN) for determining the integrity of gDNA was calculated from the electrophoretic trace on the 2200 Tape Station system according to the manufacturer’s instructions. The average value (range) of total DNA concentration in FFPE tissue and FF tissue was 322.7 (12.6 ∼ 322.9) ng/uL and 952.8 (76.0 ∼ 3756.0) ng/uL, respectively. The average value (range) of DIN in FFPE tissue and FF tissue was 2.9 (1.5 ∼ 6.4) and 5.6 (1.4 ∼ 8.4), respectively.

### Detection of EBV and *H. pylori* infection

EBV infection was detected using the Real-Q EBV quantification kit (Biosewoom, Seoul, Korea) and CFX96 real-time PCR system (Bio-Rad, USA) following the manufacturer’s recommendations.

*H. pylori* infection was detected using Giemsa stain (n=93) or PCR amplification and sequencing (n=14). Primers for PCR and sequencing were derived from a known sequence of the 23S rRNA gene (GenBank Accession No. U27270), as previously described (sense, 5'-CGT AAC TAT AACGGT CCT AAG-3', positions 2365 to 2385; antisense, 5'-TTA GCT AAC AGA AAC ATC AAG-3', positions 2635 to 2653) [41].

### Configuration of a gastric cancer-related target gene panel for Korean gastric cancer patients

The cancer panel consisted of genes based on the Mutation Analysis (MutSig 2CV v3.1) results of the Cancer Genome Atlas (TCGA) project (http://gdac.broadinstitute.org/runs/analyses_latest/reports/cancer/STAD-TP/index.html, accessed at 2015.03.30) [28, 40] and significantly mutated genes in the Asian Cancer Research Group (ACRG) cohort and SMC-2 cohort in primary gastric cancer tissues [6]. Genes associated with new targeted therapy of GC (*EGFR, ERBB2, FGFR2,* and *VEGFR2* (*KDR*)) and hereditary cancer syndromes (*CDH1, MSH2, MLH1, STK11,* and *TP53*) were also included in the cancer panel [12].

The entire length of the ROI of the NGS panel of 43 genes was 124,132 bp. To validate the performance of the NGS panel of 43 genes, NA12878 reference materials were used 7 times in 3 batches. The panel average coverage is 1,710× with 97% of targeted bases covered >20×. We downloaded the VCF file for NA12878 (https://www.ncbi.nlm.nih.gov/variation/tools/get-rm/) and then compared it to 7 variant call sets of our control reference materials (NA12878). The sensitivity and specificity of the 43 gene cancer panel were 96.4 % (95% CI: 0.941 – 0.979) and 100%, respectively.

### Targeted sequencing and data analysis

DNA fragments of matched tumor and non-tumor tissues were enriched by solution-based hybridization capture, followed by sequencing with the Illumina Hiseq2500 platform (Illumina, San Diego, CA, USA) with the 2 × 125 bp paired-end read module. gDNA was sheared using an Adaptive Focused Acoustics (AFA)™ with the Covaris Focused-ultrasonicator (Covaris, Inc., Woburn, MA, USA). The quality and quantity of sheared DNA were assessed using the Agilent 2200 Tape Station system with Agilent D1000 ScreenTape (Agilent Technologies, USA) according to the manufacturer’s instructions. Capture probes for the coding exons of 43 genes (Supplementary 1)were generated by Celemics (Seoul, Korea). Purification and clean-up of samples were performed using a DynaMag™-50 Magnet (Thermo Fisher Scientific Inc., Waltham, MA, USA) with Agencourt^®^ AMPure^®^ XP Kit (Beckman Coulter, Brea, CA, USA). NGS library amplification was performed using a KAPA Library Amplification Kit (Kapa Biosystems, Inc., Wilmington, MA, USA) according to the manufacturer’s instructions. Library preparation, hybridization, capture procedure, and sequencing on the Illumina HiSeq2500 genome analyzer were performed by Celemics according to the protocols recommended by the Celemics User Manual Ver 2.1 (http://www.celemics.com/home/).

The generated reads were trimmed and filtered by Trimmomatic [42] and then mapped against the UCSC hg19 Genome Reference Consortium Human Reference 37 (GRCh37) (http://genome.ucsc.edu/) using the Burrows-Wheeler Aligner (BWA) [43]. Picard (http://broadinstitute.github.io/picard/), SAMTools [44], and Genome Analysis Toolkit (GATK, https://www.broadinstitute.org/gatk/) [45] were used for post-processing alignments, base quality score recalibration, and short insertion/deletion (indel) realignment. After variant calling, variants were added to the annotation using ANNOVAR (http://www.openbioinformatics.org/annovar/)[46] and Variant Effect Predictor (VEP, http://asia.ensembl.org/info/docs/tools/vep/index.html). We used Varscan 2 (http://varscan.sourceforge.net) [47] for the detection of somatic SNVs and indels.

All acquired candidate variations went through post filters recommended by the authors of these tools. We extracted somatic mutations with Varscan2 and post-filtered with downstream analysis for altered allele frequency in tumors > 5%, > 50 x coverage, exonic variants, and population frequency 0.005 less than in the 1000 Genome Project (http://www.1000genomes.org), ESP6500 (http://evs.gs.washington.edu/EVS/), and Exome Aggregation Consortium (ExAC, http://exac.broadinstitute.org/). We excluded somatic variants detected >2 times in non-tumor tissue. We identified germline variations post-filtered with downstream analysis for altered allele frequency > 30%, > 50x coverage, and population frequency less than 0.01 in the 1000 Genome Project, ESP6500, and ExAC databases. These variants were present in both GC and matched non-tumor" tissue. Visual inspection of filtered calls was performed using Integrated Genomics Viewer 2.3 software (IGV; Broad Institute, Cambridge, MA, USA).

### CNV analysis

CNV analysis of the NGS panel of 43 genes was performed with dispersion and the Hidden Markov Model (HMM) method with normalized counts in NextGENe v2.4.1.2 - CNV tool (Softgenetics, State College, PA, USA). The dispersion value was automatically calculated and an HMM was used to merge multiple-exon calls and apply a priori probability. Using the coverage ratio value and the amount of noise in each region, the copy number state of each region in the sample was reported (duplication/normal/deletion) [48]. The NextGENe Viewer (SoftGenetics) was used to visualize the several large CNV calls. To validate the performance of this tool, we compared its results to Her2 immunohistochemistry (IHC) results. Fifty-four cases performed with Her2 IHC consisted of 4 positive cases (score: 3+) and 39 negative cases. We compared the Her2 IHC results and the CNV results from NextGENe-CNV analysis. The sensitivity and specificity of CNV analysis were 75.0% (95% CI: 0.194 – 0.993) and 100% (95% CI: 0.929 – 1.0), respectively.

The Infinium^®^ Global Screening Array (Illumina, San Diego, CA, USA) was performed for 69 FF tumor tissues and 22 FFPE tumor tissues according to the manufacturer’s recommendations. The hybridized arrays were scanned using the HiScan system (Illumina, San Diego, CA, USA). CNA analysis from single nucleotide polymorphism (SNP) based arrays were performed using GISTIC 2.0. [49]. To eliminate bias from copy number variable regions in healthy individuals, we analyzed CNV in a certain range, including somatic CAN-reported regions in GCs (http://www.cbioportal.org/) and dosage-sensitive regions of the genome [50].

### Microsatellite instability (MSI) assay

Microsatellite status was assessed by the mononucleotide repeat markers BAT-25, BAT-26, NR-21, NR-24, and NR-27 in tumor and corresponding non-tumor tissues [51]. The five markers were co-amplified in multiplex PCRs performed with Solg2X multiplex PCR Smart mix following the manufacturer’s recommendations. The amplified PCR products were analyzed using the ABI 3500Dx system (Applied Biosystems, Foster City, CA, USA) and GeneMarker software (SoftGenetics, PA, USA). Tumors with two or more of the five markers showing instability were judged as high-frequency MSI (MSI-H), and tumors showing instability in only one locus were classified as low-frequency MSI (MSI-L) [52].

### Molecular subtype classification and statistical analysis

As with the TCGA classification sequence [5], we also divided GC into EBV, MSI and CIN serially according to the results of EBV, MSI and SNP arrays. The remainder was then classified into the GS subgroup.

Fisher`s exact and Chi-squared tests were performed to evaluate differences in the respective proportion of several factors between subgroups. Patient follow-up periods were calculated as time between date of surgery and date of last follow-up (months). Relapse-free survival (RFS) was assessed based on the absence of loco-regional recurrence, distant metastasis, and death from any cause. GC-specific survival (GCSS) was calculated only for patients who died from any GC-related cause. Kaplan-Meier survival curves with log-rank tests were performed to compare RFS and GCSS according to AJCC stage and molecular subtype. Cox proportional hazard models were performed to assess the influence of prognostic factors on RFS. All statistical analyses were performed using SPSS 22.0 (SPSS, Chicago, IL, USA). Except for the univariate analysis, a *p* value less than 0.05 was regarded as significant.

## SUPPLEMENTARY MATERIALS TABLES





## Abbreviations

AMP: amplification
CIN: chromosomal instability
DEL: deletion
CNV: copy number variations
EBV: Epstein-Barr virus
GS: genomically stable
GC: gastric cancer
GEJ: gastroesophageal junction
H. pylori: Helicobacter pylori
MSI: microsatellite instability
Mixed: mixed type with tubular and poorly cohesive
pT stage: pathological assessment of the primary tumor
pN stage: pathological assessment of the regional lymph nodes
Uncommon: uncommon histologic variants
VAF: Variant allele frequency
VUS: a variant of unknown significance
