# Supplementary material for: Profiling cancer-associated genetic alterations and molecular classification of cancer in Korean gastric cancer patients
Source: Oncotarget. 2017 Jul 22;8(41):69888–905. doi: 10.18632/oncotarget.19435 (PMC5642524; doi:10.18632/oncotarget.19435)
Supplement: Supplementary file 1 [file oncotarget-08-69888-s001.pdf]

Profiling cancer-associated genetic alterations and molecular classification of cancer in Korean gastric cancer patients

SUPPLEMENTARY MATERIALS

Supplementary Table 1: List of 43 gastric cancer related target gene panel

ACVR1B	*ALK*	*APC*	*ARID1A*	*BCOR*	*BRAF*	*MEDAG*
C16orf74	*CBWD1*	*CCND1*	*CCNE1*	*CDH1*	*CIC*	*CR1*
CTNNB1	*EGFR*	*ERBB2*	*ERBB3*	*FBXW7*	*FGFR2*	*HLA-B*
IRF2	*JAK2*	*KRAS*	*LARP4B*	*MDM2*	*MET*	*MLH1*
MSH2	*MTOR*	*MVK*	*MYC*	*CD274*	*PDCD1LG2*	*PGM5*
PIK3CA	*PTEN*	*RHOA*	*SMAD4*	*STK11*	*TP53*	*KDR*
ZBTB20						

Supplementary Table 2: Overview of all somatic SNVs and indels identified through NGS in GC

See Supplementary File 1

Supplementary Table 3: Somatic variants of the 43 cancer panel genes in 107 Korean gastric cancers and TCGA data

Gene	In this study (n=107)		TCGA, nature 2014 (n=289)	
	No of case	Case (%)	No of case	Case (%)
TP53	41	38.3%	138	47.8%
ARID1A	39	36.4%	90	31.1%
CR1	16	15.0%	21	7.3%
APC	12	11.2%	42	14.5%
BCOR	12	11.2%	21	7.3%
CDH1	11	10.3%	29	10.0%
CIC	10	9.3%	26	9.0%
PIK3CA	10	9.3%	57	19.7%
ERBB3	9	8.4%	31	10.7%
RHOA	9	8.4%	16	5.5%
ERBB2	8	7.5%	14	4.8%
CCND1	7	6.5%	1	0.3%
FBXW7	7	6.5%	27	9.3%
ALK	6	5.6%	12	4.2%
KRAS	6	5.6%	28	9.7%
MTOR	6	5.6%	23	8.0%
CTNNB1	5	4.7%	19	6.6%
EGFR	5	4.7%	15	5.2%
HLA-B	5	4.7%	20	6.9%
MSH2	5	4.7%	5	1.7%
PGM5	5	4.7%	25	8.7%
ZBTB20	5	4.7%	28	9.7%
IRF2	4	3.7%	15	5.2%
KDR	4	3.7%	13	4.5%
LARP4B	4	3.7%	27	9.3%
MVK	4	3.7%	13	4.5%
BRAF	3	2.8%	16	5.5%
PTEN	3	2.8%	23	8.0%
ACVR1B	2	1.9%	11	3.8%
CBWD1	2	1.9%	1	0.3%
FGFR2	2	1.9%	12	4.2%
JAK2	2	1.9%	12	4.2%
MEDAG	2	1.9%	7	2.4%
MLH1	2	1.9%	6	2.1%
SMAD4	2	1.9%	24	8.3%
STK11	2	1.9%	3	1.0%
CD274	1	0.9%	3	1.0%
MDM2	1	0.9%	15	5.2%
MYC	1	0.9%	5	1.7%
C16orf74	0	0.0%	2	0.7%
CCNE1	0	0.0%	5	1.7%
MET	0	0.0%	6	2.1%
PDCD1LG2	0	0.0%	1	0.3%
