# Supplementary material for: Profiling cancer-associated genetic alterations and molecular classification of cancer in Korean gastric cancer patients
Source: Oncotarget. 2017 Jul 22;8(41):69888–905. doi: 10.18632/oncotarget.19435 (PMC5642524; doi:10.18632/oncotarget.19435)
Supplement: Supplementary file 2 [file oncotarget-08-69888-s002.docx]

**Supplementary Table 2: Overview of all somatic SNVs and indels identified through NGS in GC**

| **Sample** | **chr** | | **pos** | **Ref** | **Alt** | **Coverage** | **Alter_Allel_freq (%)** | **gene_name** | **Effect** | **hgvs_transcript** | **hgvs_protein** |
| --- | --- | --- | --- | --- | --- | --- | --- | --- | --- | --- | --- |
| SKW2 | 17 | 7577124 | | C | T | 685 | 9% | TP53 | missense_variant | ENST00000269305.4:c.814G>T | ENSP00000269305.4:p.Val272Leu |
| SKW5 | 9 | 121567 | | T | A | 359 | 20% | CBWD1 | missense_variant | ENST00000314367.10:c.980A>T | ENSP00000323433.10:p.Asn327Ile |
| SKW5 | 19 | 42794626 | | G | A | 77 | 5% | CIC | missense_variant | ENST00000160740.3:c.1706G>A | ENSP00000160740.3:p.Gly569Asp |
| SKW5 | 1 | 207787753 | | C | T | 354 | 13% | CR1 | stop_gained | ENST00000367049.4:c.6580C>T | ENSP00000356016.4:p.Arg2194Ter |
| SKW6 | 17 | 7578406 | | C | T | 294 | 44% | TP53 | missense_variant | ENST00000269305.4:c.524G>A | ENSP00000269305.4:p.Arg175His |
| SKW13 | 1 | 27101441 | | C | T | 80 | 5% | ARID1A | missense_variant | ENST00000324856.7:c.4723C>T | ENSP00000320485.7:p.Pro1575Ser |
| SKW13 | 1 | 27105930 | | TG | T | 179 | 5% | ARID1A | frameshift_variant | ENST00000324856.7:c.5548delG | ENSP00000320485.7:p.Asp1850ThrfsTer33 |
| SKW13 | 9 | 122002 | | C | A | 85 | 6% | CBWD1 | missense_variant | ENST00000314367.10:c.932G>T | ENSP00000323433.10:p.Trp311Leu |
| SKW13 | 19 | 42792038 | | C | A | 96 | 6% | CIC | missense_variant | ENST00000160740.3:c.842C>A | ENSP00000160740.3:p.Ala281Asp |
| SKW13 | 4 | 185340680 | | G | T | 130 | 5% | IRF2 | missense_variant | ENST00000393593.3:c.130C>A | ENSP00000377218.3:p.His44Asn |
| SKW13 | 4 | 185340682 | | C | A | 126 | 6% | IRF2 | missense_variant | ENST00000393593.3:c.128G>T | ENSP00000377218.3:p.Arg43Ile |
| SKW13 | 2 | 47635593 | | G | T | 149 | 7% | MSH2 | missense_variant | ENST00000233146.2:c.265G>T | ENSP00000233146.2:p.Val89Leu |
| SKW15 | 17 | 7578236 | | A | G | 973 | 41% | TP53 | missense_variant | ENST00000269305.4:c.613T>C | ENSP00000269305.4:p.Tyr205His |
| SKW16 | 1 | 207787753 | | C | T | 854 | 7% | CR1 | stop_gained | ENST00000367049.4:c.6580C>T | ENSP00000356016.4:p.Arg2194Ter |
| SKW12 | 1 | 27100181 | | CGCA | C | 520 | 5% | ARID1A | In_Frame_variant | ENST00000324856.7:c.3999_4001del GCA | ENSP00000320485.7:p.Gln1334del |
| SKW12 | 16 | 68771347 | | CGCT | C | 61 | 8% | CDH1 | In_Frame_variant | ENST00000261769.5:c.44_46delTGC | ENSP00000261769.4:p.Leu15del |
| SKW12 | 8 | 128750639 | | C | T | 618 | 9% | MYC | missense_variant | ENST00000259523.6:c.131C>T | ENSP00000259523.6:p.Ala44Val |
| SKW17 | 1 | 207787753 | | C | T | 259 | 12% | CR1 | stop_gained | ENST00000367049.4:c.6580C>T | ENSP00000356016.4:p.Arg2194Ter |
| SKW17 | 10 | 876899 | | T | A | 99 | 6% | LARP4B | stop_gained | ENST00000316157.3:c.769A>T | ENSP00000326128.3:p.Lys257Ter |
| SKW17 | 1 | 11190648 | | T | C | 188 | 8% | MTOR | missense_variant | ENST00000361445.4:c.5551A>G | ENSP00000354558.4:p.Ser1851Gly |
| SKW17 | 19 | 1220383 | | A | G | 73 | 5% | STK11 | missense_variant | ENST00000326873.7:c.476A>G | ENSP00000324856.6:p.Gln159Arg |
| SKW18 | 1 | 27057936 | | GC | G | 750 | 9% | ARID1A | frameshift_variant | ENST00000324856.7:c.1650delC | ENSP00000320485.7:p.Tyr551ThrfsTer68 |
| SKW18 | 17 | 7577022 | | G | A | 847 | 11% | TP53 | stop_gained | ENST00000269305.4:c.916C>T | ENSP00000269305.4:p.Arg306Ter |
| SKW18 | 12 | 25398281 | | C | T | 780 | 10% | KRAS | missense_variant | ENST00000256078.4:c.38G>A | ENSP00000256078.4:p.Gly13Asp |
| SKW18 | 13 | 31495945 | | GC | G | 373 | 6% | MEDAG | frameshift_variant | ENST00000380482.4:c.750delC | ENSP00000369849.4:p.Ser250ArgfsTer10 |
| SKW18 | 3 | 37061839 | | AC | A | 477 | 11% | MLH1 | frameshift_variant | ENST00000231790.2:c.927delC | ENSP00000231790.2:p.Thr310GlnfsTer57 |
| SKW18 | 3 | 178952085 | | A | G | 615 | 9% | PIK3CA | missense_variant | ENST00000263967.3:c.3140A>G | ENSP00000263967.3:p.His1047Arg |
| SKW20 | 1 | 27087467 | | T | G | 219 | 8% | ARID1A | missense_variant | ENST00000324856.7:c.2041T>G | ENSP00000320485.7:p.Phe681Val |
| SKW20 | 1 | 207787753 | | C | T | 289 | 15% | CR1 | stop_gained | ENST00000367049.4:c.6580C>T | ENSP00000356016.4:p.Arg2194Ter |
| SKW20 | 6 | 31323094 | | C | T | 145 | 8% | HLA-B | missense_variant | ENST00000412585.2:c.895G>A | ENSP00000399168.2:p.Glu299Lys |
| SKW20 | 1 | 11190702 | | T | C | 207 | 5% | MTOR | missense_variant | ENST00000361445.4:c.5497A>G | ENSP00000354558.4:p.Thr1833Ala |
| SKW25 | 4 | 153332725 | | C | A | 62 | 6% | FBXW7 | missense_variant | ENST00000281708.4:c.231G>T | ENSP00000281708.3:p.Leu77Phe |
| SKW25 | 4 | 153332588 | | TCA | T | 64 | 6% | FBXW7 | frameshift_variant | ENST00000281708.4:c.366_367delTG | ENSP00000281708.3:p.Ser122ArgfsTer4 |
| SKW21 | X | 39913252 | | TG | T | 79 | 5% | BCOR | frameshift_variant | ENST00000342274.4:c.4760delC | ENSP00000345923.4:p.Pro1587GlnfsTer53 |
| SKW21 | 6 | 31324602 | | T | A | 64 | 8% | HLA-B | missense_variant | ENST00000412585.2:c.206A>T | ENSP00000399168.2:p.Glu69Val |
| SKW23 | 1 | 27106732 | | C | T | 729 | 19% | ARID1A | stop_gained | ENST00000324856.7:c.6343C>T | ENSP00000320485.7:p.Gln2115Ter |
| SKW23 | 17 | 7577547 | | C | A | 295 | 12% | TP53 | missense_variant | ENST00000269305.4:c.734G>T | ENSP00000269305.4:p.Gly245Val |
| SKW1 | 17 | 7576855 | | G | A | 678 | 42% | TP53 | stop_gained | ENST00000269305.4:c.991C>T | ENSP00000269305.4:p.Gln331Ter |
| SKW1 | 11 | 69465987 | | AGAG | A | 201 | 5% | CCND1 | In_Frame_variant | ENST00000227507.2:c.839_841delAGG | ENSP00000227507.2:p.Glu280del |
| SKW38 | 2 | 29451783 | | AC | A | 64 | 6% | ALK | frameshift_variant | ENST00000389048.3:c.2781delG | ENSP00000373700.3:p.Cys928AlafsTer11 |
| SKW38 | 17 | 7578527 | | A | G | 157 | 29% | TP53 | missense_variant | ENST00000269305.4:c.403T>C | ENSP00000269305.4:p.Cys135Arg |
| SKW38 | 1 | 207787753 | | C | T | 233 | 16% | CR1 | stop_gained | ENST00000367049.4:c.6580C>T | ENSP00000356016.4:p.Arg2194Ter |
| SKW38 | 3 | 49412913 | | G | A | 177 | 23% | RHOA | missense_variant | ENST00000418115.1:c.110C>T | ENSP00000400175.1:p.Thr37Ile |
| SKW37 | 1 | 27057658 | | C | T | 586 | 14% | ARID1A | stop_gained | ENST00000324856.7:c.1366C>T | ENSP00000320485.7:p.Gln456Ter |
| SKW37 | 1 | 27105897 | | G | GTT | 879 | 18% | ARID1A | frameshift_variant | ENST00000324856.7:c.5510_5511dupTT | ENSP00000320485.7:p.Asp1838LeufsTer46 |
| SKW36 | 2 | 30142909 | | G | A | 352 | 10% | ALK | missense_variant | ENST00000389048.3:c.617C>T | ENSP00000373700.3:p.Ala206Val |
| SKW26 | 1 | 27023140 | | TGGC | T | 55 | 7% | ARID1A | In_Frame_variant | ENST00000324856.7:c.258_260delCGG | ENSP00000320485.7:p.Gly87del |
| SKW26 | 1 | 27101116 | | AC | A | 977 | 14% | ARID1A | frameshift_variant | ENST00000324856.7:c.4403delC | ENSP00000320485.7:p.Pro1468LeufsTer13 |
| SKW26 | X | 39933399 | | GC | G | 648 | 32% | BCOR | frameshift_variant | ENST00000342274.4:c.1199delG | ENSP00000345923.4:p.Gly400AlafsTer42 |
| SKW26 | 7 | 55231495 | | G | A | 745 | 19% | EGFR | missense_variant | ENST00000275493.2:c.1701G>A | ENSP00000275493.2:p.Met567Ile |
| SKW26 | 12 | 56486562 | | C | A | 1305 | 14% | ERBB3 | missense_variant | ENST00000267101.3:c.1141C>A | ENSP00000267101.3:p.Pro381Thr |
| SKW26 | 12 | 56489493 | | T | A | 2582 | 17% | ERBB3 | missense_variant | ENST00000267101.3:c.1958T>A | ENSP00000267101.3:p.Val653Glu |
| SKW26 | 10 | 123263394 | | C | T | 1176 | 16% | FGFR2 | missense_variant | ENST00000336553.6:c.1076G>A | ENSP00000337665.6:p.Arg359His |
| SKW26 | 12 | 25398281 | | C | T | 2457 | 6% | KRAS | missense_variant | ENST00000256078.4:c.38G>A | ENSP00000256078.4:p.Gly13Asp |
| SKW26 | 2 | 47639587 | | GA | G | 1023 | 14% | MSH2 | frameshift_variant | ENST00000233146.2:c.687delA | ENSP00000233146.2:p.Ala230LeufsTer16 |
| SKW26 | 12 | 110034311 | | G | A | 239 | 15% | MVK | missense_variant | ENST00000228510.3:c.1120G>A | ENSP00000228510.3:p.Ala374Thr |
| SKW26 | 19 | 1221313 | | GC | G | 468 | 15% | STK11 | frameshift_variant | ENST00000326873.7:c.842delC | ENSP00000324856.6:p.Pro281ArgfsTer6 |
| SKW26 | 3 | 114058002 | | AG | A | 1296 | 16% | ZBTB20 | frameshift_variant | ENST00000357258.3:c.1856delC | ENSP00000349803.3:p.Pro619LeufsTer43 |
| SKW27 | 1 | 27101401 | | GC | G | 183 | 15% | ARID1A | frameshift_variant | ENST00000324856.7:c.4689delC | ENSP00000320485.7:p.Met1564Ter |
| SKW27 | 16 | 68772281 | | C | T | 54 | 7% | CDH1 | missense_variant | ENST00000261769.5:c.130C>T | ENSP00000261769.4:p.Arg44Cys |
| SKW27 | 9 | 70972209 | | G | A | 71 | 6% | PGM5 | missense_variant | ENST00000396392.1:c.166G>A | ENSP00000379674.1:p.Asp56Asn |
| SKW29 | 19 | 42791048 | | C | T | 498 | 19% | CIC | missense_variant | ENST00000160740.3:c.193C>T | ENSP00000160740.3:p.Pro65Ser |
| SKW29 | 17 | 37868208 | | C | T | 643 | 24% | ERBB2 | missense_variant | ENST00000269571.5:c.929C>T | ENSP00000269571.4:p.Ser310Phe |
| SKW29 | 12 | 25398284 | | C | T | 929 | 21% | KRAS | missense_variant | ENST00000256078.4:c.35G>A | ENSP00000256078.4:p.Gly12Asp |
| SKW29 | 3 | 178936094 | | C | A | 657 | 13% | PIK3CA | missense_variant | ENST00000263967.3:c.1636C>A | ENSP00000263967.3:p.Gln546Lys |
| SKW29 | 18 | 48575191 | | A | T | 768 | 21% | SMAD4 | missense_variant | ENST00000342988.3:c.385A>T | ENSP00000341551.3:p.Asn129Tyr |
| SKW43 | 17 | 7577539 | | G | A | 444 | 7% | TP53 | missense_variant | ENST00000269305.4:c.742C>T | ENSP00000269305.4:p.Arg248Trp |
| SKW43 | 16 | 68771347 | | CGCT | C | 79 | 5% | CDH1 | In_Frame_variant | ENST00000261769.5:c.44_46delTGC | ENSP00000261769.4:p.Leu15del |
| SKW43 | 1 | 207780640 | | G | A | 1110 | 9% | CR1 | missense_variant | ENST00000367049.4:c.5855G>A | ENSP00000356016.4:p.Gly1952Asp |
| SKW42 | 1 | 27057825 | | CCA | C | 1355 | 9% | ARID1A | frameshift_variant | ENST00000324856.7:c.1534_1535delCA | ENSP00000320485.7:p.Gln512ValfsTer110 |
| SKW42 | X | 39923086 | | G | GT | 583 | 26% | BCOR | frameshift_variant | ENST00000342274.4:c.3519dupA | ENSP00000345923.4:p.Gln1174ThrfsTer8 |
| SKW42 | 11 | 69465987 | | AGAG | A | 116 | 5% | CCND1 | In_Frame_variant | ENST00000227507.2:c.839_841delAGG | ENSP00000227507.2:p.Glu280del |
| SKW42 | 7 | 55249007 | | G | A | 634 | 12% | EGFR | missense_variant | ENST00000275493.2:c.2305G>A | ENSP00000275493.2:p.Val769Met |
| SKW42 | 10 | 890938 | | GT | G | 1127 | 13% | LARP4B | frameshift_variant | ENST00000316157.3:c.487delA | ENSP00000326128.3:p.Thr163HisfsTer47 |
| SKW42 | 2 | 47639648 | | CA | C | 785 | 11% | MSH2 | frameshift_variant | ENST00000233146.2:c.746delA | ENSP00000233146.2:p.Lys249ArgfsTer5 |
| SKW41 | 1 | 27097687 | | GA | G | 2637 | 29% | ARID1A | frameshift_variant | ENST00000324856.7:c.3281delA | ENSP00000320485.7:p.Lys1094SerfsTer67 |
| SKW41 | 1 | 27100175 | | A | AC | 1439 | 24% | ARID1A | frameshift_variant | ENST00000324856.7:c.3977dupC | ENSP00000320485.7:p.Gln1327AlafsTer11 |
| SKW41 | X | 39914723 | | G | A | 2516 | 34% | BCOR | stop_gained | ENST00000342274.4:c.4537C>T | ENSP00000345923.4:p.Arg1513Ter |
| SKW41 | 19 | 42794440 | | GC | G | 821 | 27% | CIC | frameshift_variant | ENST00000160740.3:c.1526delC | ENSP00000160740.3:p.Pro509HisfsTer14 |
| SKW41 | 19 | 42797375 | | GC | G | 648 | 36% | CIC | frameshift_variant | ENST00000160740.3:c.3737delC | ENSP00000160740.3:p.Pro1246HisfsTer54 |
| SKW41 | 1 | 207787753 | | C | T | 4083 | 17% | CR1 | stop_gained | ENST00000367049.4:c.6580C>T | ENSP00000356016.4:p.Arg2194Ter |
| SKW41 | 1 | 207787831 | | G | T | 3853 | 7% | CR1 | stop_gained | ENST00000367049.4:c.6658G>T | ENSP00000356016.4:p.Glu2220Ter |
| SKW41 | 17 | 37884008 | | C | T | 551 | 34% | ERBB2 | missense_variant | ENST00000269571.5:c.3479C>T | ENSP00000269571.4:p.Ala1160Val |
| SKW41 | 4 | 153249384 | | C | T | 1769 | 28% | FBXW7 | missense_variant | ENST00000263981.5:c.1154G>A | ENSP00000263981.4:p.Arg385His |
| SKW41 | 6 | 31324207 | | AG | A | 193 | 6% | HLA-B | frameshift_variant | ENST00000412585.2:c.355delC | ENSP00000399168.2:p.Leu119SerfsTer32 |
| SKW41 | 6 | 31324508 | | C | CT | 600 | 5% | HLA-B | frameshift_variant | ENST00000412585.2:c.299dupA | ENSP00000399168.2:p.Ser101GlufsTer38 |
| SKW41 | 12 | 25398281 | | C | T | 3172 | 18% | KRAS | missense_variant | ENST00000256078.4:c.38G>A | ENSP00000256078.4:p.Gly13Asp |
| SKW41 | 12 | 69230470 | | T | C | 3254 | 47% | MDM2 | missense_variant | ENST00000258148.7:c.694T>C | ENSP00000258148.7:p.Tyr232His |
| SKW41 | 9 | 70993121 | | C | T | 3716 | 6% | PGM5 | stop_gained | ENST00000396392.1:c.268C>T | ENSP00000379674.1:p.Arg90Ter |
| SKW41 | 9 | 70993145 | | A | G | 3607 | 12% | PGM5 | missense_variant | ENST00000396392.1:c.292A>G | ENSP00000379674.1:p.Ile98Val |
| SKW41 | 3 | 178952085 | | A | G | 1997 | 21% | PIK3CA | missense_variant | ENST00000263967.3:c.3140A>G | ENSP00000263967.3:p.His1047Arg |
| SKW41 | 3 | 114058002 | | AG | A | 1623 | 6% | ZBTB20 | frameshift_variant | ENST00000357258.3:c.1856delC | ENSP00000349803.3:p.Pro619LeufsTer43 |
| SKW31 | 2 | 30143407 | | G | A | 92 | 5% | ALK | missense_variant | ENST00000389048.3:c.119C>T | ENSP00000373700.3:p.Pro40Leu |
| SKW31 | 1 | 27105930 | | TG | T | 1752 | 20% | ARID1A | frameshift_variant | ENST00000324856.7:c.5548delG | ENSP00000320485.7:p.Asp1850ThrfsTer33 |
| SKW31 | 19 | 42791228 | | GC | G | 270 | 6% | CIC | frameshift_variant | ENST00000160740.3:c.293delC | ENSP00000160740.3:p.Pro98LeufsTer107 |
| SKW31 | 17 | 37879658 | | G | A | 1353 | 15% | ERBB2 | missense_variant | ENST00000269571.5:c.2033G>A | ENSP00000269571.4:p.Arg678Gln |
| SKW31 | 12 | 56493477 | | C | T | 1215 | 9% | ERBB3 | missense_variant | ENST00000267101.3:c.2885C>T | ENSP00000267101.3:p.Ala962Val |
| SKW31 | 10 | 89717769 | | T | TA | 1885 | 26% | PTEN | frameshift_variant | ENST00000371953.3:c.800dupA | ENSP00000361021.3:p.Asp268GlyfsTer30 |
| SKW33 | 17 | 7578535 | | T | C | 720 | 11% | TP53 | missense_variant | ENST00000269305.4:c.395A>G | ENSP00000269305.4:p.Lys132Arg |
| SKW33 | 11 | 69465987 | | AGAG | A | 111 | 5% | CCND1 | In_Frame_variant | ENST00000227507.2:c.839_841delAGG | ENSP00000227507.2:p.Glu280del |
| SKW33 | 12 | 56494920 | | GCATCAGAGT | G | 358 | 6% | ERBB3 | In_Frame_variant | ENST00000267101.3:c.3286_3294delTCATCAGAG | ENSP00000267101.3:p.Ser1096_Glu1098del |
| SKW45 | 1 | 27105930 | | TG | T | 625 | 8% | ARID1A | frameshift_variant | ENST00000324856.7:c.5548delG | ENSP00000320485.7:p.Asp1850ThrfsTer33 |
| SKW45 | 17 | 7577548 | | C | T | 282 | 13% | TP53 | missense_variant | ENST00000269305.4:c.733G>A | ENSP00000269305.4:p.Gly245Ser |
| SKW45 | 7 | 140482926 | | AG | A | 770 | 8% | BRAF | frameshift_variant | ENST00000288602.6:c.1208delC | ENSP00000288602.6:p.Pro403LeufsTer8 |
| SKW45 | 11 | 69465987 | | AGAG | A | 73 | 8% | CCND1 | In_Frame_variant | ENST00000227507.2:c.839_841delAGG | ENSP00000227507.2:p.Glu280del |
| SKW45 | 16 | 68845649 | | G | A | 667 | 9% | CDH1 | missense_variant | ENST00000261769.5:c.895G>A | ENSP00000261769.4:p.Ala299Thr |
| SKW45 | 16 | 68771347 | | CGCT | C | 76 | 8% | CDH1 | In_Frame_variant | ENST00000261769.5:c.44_46delTGC | ENSP00000261769.4:p.Leu15del |
| SKW45 | 19 | 42791265 | | G | A | 168 | 11% | CIC | missense_variant | ENST00000160740.3:c.325G>A | ENSP00000160740.3:p.Gly109Arg |
| SKW45 | 19 | 42799097 | | GC | G | 205 | 7% | CIC | frameshift_variant | ENST00000160740.3:c.4580delC | ENSP00000160740.3:p.Pro1527LeufsTer91 |
| SKW45 | 3 | 41278163 | | GCT | G | 746 | 7% | CTNNB1 | frameshift_variant | ENST00000349496.5:c.2046_2047delCT | ENSP00000344456.5:p.Phe683GlnfsTer9 |
| SKW45 | 7 | 55229242 | | G | T | 383 | 10% | EGFR | missense_variant | ENST00000275493.2:c.1549G>T | ENSP00000275493.2:p.Gly517Cys |
| SKW45 | 17 | 37881332 | | G | A | 286 | 14% | ERBB2 | missense_variant | ENST00000269571.5:c.2524G>A | ENSP00000269571.4:p.Val842Ile |
| SKW45 | 12 | 56478854 | | G | A | 638 | 20% | ERBB3 | missense_variant | ENST00000267101.3:c.310G>A | ENSP00000267101.3:p.Val104Met |
| SKW45 | 4 | 55976714 | | G | T | 387 | 5% | KDR | missense_variant | ENST00000263923.4:c.1111C>A | ENSP00000263923.4:p.Leu371Ile |
| SKW45 | 2 | 47705561 | | T | TA | 589 | 10% | MSH2 | frameshift_variant | ENST00000233146.2:c.2362dupA | ENSP00000233146.2:p.Thr788AsnfsTer11 |
| SKW45 | 2 | 47707887 | | TA | T | 725 | 12% | MSH2 | frameshift_variant | ENST00000233146.2:c.2513delA | ENSP00000233146.2:p.Lys838SerfsTer3 |
| SKW45 | 1 | 11182095 | | G | A | 360 | 6% | MTOR | missense_variant | ENST00000361445.4:c.6751C>T | ENSP00000354558.4:p.Arg2251Trp |
| SKW45 | 3 | 49412914 | | T | C | 617 | 15% | RHOA | missense_variant | ENST00000418115.1:c.109A>G | ENSP00000400175.1:p.Thr37Ala |
| SKW44 | 1 | 207787753 | | C | T | 193 | 19% | CR1 | stop_gained | ENST00000367049.4:c.6580C>T | ENSP00000356016.4:p.Arg2194Ter |
| SKW44 | 4 | 185340688 | | G | C | 147 | 5% | IRF2 | missense_variant | ENST00000393593.3:c.122C>G | ENSP00000377218.3:p.Ala41Gly |
| YMC69 | 5 | 112179000 | | C | G | 238 | 39% | APC | stop_gained | ENST00000257430.4:c.7709C>G | ENSP00000257430.4:p.Ser2570Ter |
| YMC69 | 3 | 178936091 | | G | A | 201 | 9% | PIK3CA | missense_variant | ENST00000263967.3:c.1633G>A | ENSP00000263967.3:p.Glu545Lys |
| YMC69 | 17 | 7577507 | | T | G | 198 | 48% | TP53 | missense_variant | ENST00000269305.4:c.774A>C | ENSP00000269305.4:p.Glu258Asp |
| YMC69 | 17 | 7577529 | | A | G | 217 | 46% | TP53 | missense_variant | ENST00000269305.4:c.752T>C | ENSP00000269305.4:p.Ile251Thr |
| YMC63 | 5 | 112179566 | | C | T | 706 | 17% | APC | missense_variant | ENST00000257430.4:c.8275C>T | ENSP00000257430.4:p.Arg2759Cys |
| YMC63 | 9 | 5463099 | | T | A | 527 | 26% | CD274 | missense_variant | ENST00000381573.4:c.318T>A | ENSP00000370985.4:p.His106Gln |
| YMC63 | 16 | 68849539 | | AT | A | 665 | 5% | CDH1 | frameshift_variant | ENST00000261769.5:c.1443delT | ENSP00000261769.4:p.Asn481LysfsTer41 |
| YMC63 | 3 | 41266113 | | C | G | 498 | 35% | CTNNB1 | missense_variant | ENST00000349496.5:c.110C>G | ENSP00000344456.5:p.Ser37Cys |
| YMC62 | 1 | 27100181 | | CGCA | C | 357 | 6% | ARID1A | In_Frame_variant | ENST00000324856.7:c.3999_4001del GCA | ENSP00000320485.7:p.Gln1334del |
| YMC68 | 1 | 27100181 | | CGCA | C | 298 | 7% | ARID1A | In_Frame_variant | ENST00000324856.7:c.3999_4001del GCA | ENSP00000320485.7:p.Gln1334del |
| YMC68 | X | 39923086 | | G | GT | 248 | 8% | BCOR | frameshift_variant | ENST00000342274.4:c.3519dupA | ENSP00000345923.4:p.Gln1174ThrfsTer8 |
| YMC68 | 1 | 207696972 | | AC | A | 482 | 7% | CR1 | frameshift_variant | ENST00000367049.4:c.510delC | ENSP00000356016.4:p.Thr171ProfsTer21 |
| YMC68 | 17 | 37879658 | | G | A | 753 | 5% | ERBB2 | missense_variant | ENST00000269571.5:c.2033G>A | ENSP00000269571.4:p.Arg678Gln |
| YMC68 | 17 | 37881332 | | G | A | 344 | 6% | ERBB2 | missense_variant | ENST00000269571.5:c.2524G>A | ENSP00000269571.4:p.Val842Ile |
| YMC68 | 10 | 890938 | | GT | G | 235 | 6% | LARP4B | frameshift_variant | ENST00000316157.3:c.487delA | ENSP00000326128.3:p.Thr163HisfsTer47 |
| YMC61 | 1 | 27100181 | | CGCA | C | 466 | 6% | ARID1A | In_Frame_variant | ENST00000324856.7:c.3999_4001del GCA | ENSP00000320485.7:p.Gln1334del |
| YMC61 | 17 | 7579432 | | AG | A | 215 | 7% | TP53 | frameshift_variant | ENST00000269305.4:c.254delC | ENSP00000269305.4:p.Pro85LeufsTer38 |
| YMC59 | 12 | 56481922 | | G | A | 622 | 16% | ERBB3 | missense_variant | ENST00000267101.3:c.850G>A | ENSP00000267101.3:p.Gly284Arg |
| YMC59 | 17 | 7574003 | | G | A | 254 | 13% | TP53 | stop_gained | ENST00000269305.4:c.1024C>T | ENSP00000269305.4:p.Arg342Ter |
| YMC58 | 16 | 68842751 | | TGTAA | T | 382 | 22% | CDH1 | splicing_donor site_variant | ENST00000261769.5:c.687+4_687+7delAGTA | - |
| YMC58 | 3 | 41274899 | | G | A | 224 | 5% | CTNNB1 | stop_gained | ENST00000349496.5:c.1149G>A | ENSP00000344456.5:p.Trp383Ter |
| YMC58 | 7 | 55248986 | | G | A | 127 | 15% | EGFR | missense_variant | ENST00000275493.2:c.2284G>A | ENSP00000275493.2:p.Glu762Lys |
| YMC58 | 3 | 49413010 | | G | A | 306 | 7% | RHOA | missense_variant | ENST00000418115.1:c.13C>T | ENSP00000400175.1:p.Arg5Trp |
| YMC58 | 17 | 7578212 | | G | A | 684 | 7% | TP53 | stop_gained | ENST00000269305.4:c.637C>T | ENSP00000269305.4:p.Arg213Ter |
| YMC57 | 1 | 27092833 | | G | T | 837 | 9% | ARID1A | stop_gained | ENST00000324856.7:c.2854G>T | ENSP00000320485.7:p.Gly952Ter |
| YMC57 | 11 | 69465987 | | AGAG | A | 76 | 5% | CCND1 | In_Frame_variant | ENST00000227507.2:c.839_841delAGG | ENSP00000227507.2:p.Glu280del |
| YMC56 | 1 | 27100181 | | CGCA | C | 251 | 6% | ARID1A | In_Frame_variant | ENST00000324856.7:c.3999_4001del GCA | ENSP00000320485.7:p.Gln1334del |
| YMC56 | 17 | 7577508 | | T | C | 177 | 6% | TP53 | missense_variant | ENST00000269305.4:c.773A>G | ENSP00000269305.4:p.Glu258Gly |
| YMC55 | 5 | 112176020 | | G | T | 411 | 47% | APC | stop_gained | ENST00000257430.4:c.4729G>T | ENSP00000257430.4:p.Glu1577Ter |
| YMC55 | 17 | 7578413 | | C | T | 255 | 48% | TP53 | missense_variant | ENST00000269305.4:c.517G>T | ENSP00000269305.4:p.Val173Leu |
| YMC54 | 1 | 27023140 | | TGGC | T | 53 | 6% | ARID1A | In_Frame_variant | ENST00000324856.7:c.258_260delCGG | ENSP00000320485.7:p.Gly87del |
| YMC54 | 1 | 27100181 | | CGCA | C | 663 | 5% | ARID1A | In_Frame_variant | ENST00000324856.7:c.3999_4001del GCA | ENSP00000320485.7:p.Gln1334del |
| YMC54 | 17 | 7578550 | | G | T | 498 | 54% | TP53 | missense_variant | ENST00000269305.4:c.380C>A | ENSP00000269305.4:p.Ser127Tyr |
| YMC53 | 17 | 7578203 | | C | A | 969 | 13% | TP53 | missense_variant | ENST00000269305.4:c.646G>T | ENSP00000269305.4:p.Val216Leu |
| YMC51 | 1 | 27100181 | | CGCA | C | 406 | 5% | ARID1A | In_Frame_variant | ENST00000324856.7:c.3999_4001del GCA | ENSP00000320485.7:p.Gln1334del |
| YMC51 | 1 | 27105930 | | TG | T | 722 | 32% | ARID1A | frameshift_variant | ENST00000324856.7:c.5548delG | ENSP00000320485.7:p.Asp1850ThrfsTer33 |
| YMC51 | 3 | 41277260 | | C | A | 355 | 20% | CTNNB1 | missense_variant | ENST00000349496.5:c.1729C>A | ENSP00000344456.5:p.Leu577Ile |
| YMC51 | 4 | 153273635 | | A | AT | 582 | 33% | FBXW7 | frameshift_variant | ENST00000263981.5:c.247dupA | ENSP00000263981.4:p.Met83AsnfsTer15 |
| YMC51 | 4 | 55955595 | | C | G | 524 | 27% | KDR | missense_variant | ENST00000263923.4:c.3350G>C | ENSP00000263923.4:p.Arg1117Thr |
| YMC51 | 10 | 909737 | | C | T | 667 | 35% | LARP4B | missense_variant | ENST00000316157.3:c.376G>A | ENSP00000326128.3:p.Ala126Thr |
| YMC51 | 3 | 37070348 | | AC | A | 819 | 32% | MLH1 | frameshift_variant | ENST00000231790.2:c.1489delC | ENSP00000231790.2:p.Arg497GlyfsTer11 |
| YMC51 | 17 | 7577093 | | C | T | 655 | 23% | TP53 | missense_variant | ENST00000269305.4:c.845G>A | ENSP00000269305.4:p.Arg282Gln |
| YMC51 | 17 | 7572962 | | GT | G | 338 | 19% | TP53 | frameshift_variant | ENST00000269305.4:c.1146delA | ENSP00000269305.4:p.Lys382AsnfsTer40 |
| YMC51 | 3 | 114058002 | | AG | A | 315 | 30% | ZBTB20 | frameshift_variant | ENST00000357258.3:c.1856delC | ENSP00000349803.3:p.Pro619LeufsTer43 |
| YMC50 | 1 | 27023715 | | TG | T | 172 | 13% | ARID1A | frameshift_variant | ENST00000324856.7:c.827delG | ENSP00000320485.7:p.Gly276GlufsTer87 |
| YMC50 | 1 | 27097621 | | CA | C | 737 | 22% | ARID1A | frameshift_variant | ENST00000324856.7:c.3216delA | ENSP00000320485.7:p.Lys1072AsnfsTer21 |
| YMC50 | 1 | 27105930 | | T | TG | 661 | 23% | ARID1A | frameshift_variant | ENST00000324856.7:c.5548delG | ENSP00000320485.7:p.Asp1850ThrfsTer33 |
| YMC50 | X | 39923086 | | G | GT | 673 | 20% | BCOR | frameshift_variant | ENST00000342274.4:c.3519dupA | ENSP00000345923.4:p.Gln1174ThrfsTer8 |
| YMC50 | 1 | 207782716 | | T | C | 592 | 21% | CR1 | missense_variant | ENST00000367049.4:c.5978T>C | ENSP00000356016.4:p.Val1993Ala |
| YMC50 | 7 | 55240761 | | C | T | 186 | 19% | EGFR | stop_gained | ENST00000275493.2:c.2005C>T | ENSP00000275493.2:p.Arg669Ter |
| YMC50 | 12 | 56478854 | | G | A | 680 | 25% | ERBB3 | missense_variant | ENST00000267101.3:c.310G>A | ENSP00000267101.3:p.Val104Met |
| YMC50 | 12 | 56489582 | | C | T | 1226 | 23% | ERBB3 | missense_variant | ENST00000267101.3:c.2047C>T | ENSP00000267101.3:p.Arg683Trp |
| YMC50 | 4 | 153332496 | | C | T | 1394 | 22% | FBXW7 | missense_variant | ENST00000281708.4:c.460G>A | ENSP00000281708.3:p.Val154Ile |
| YMC50 | 12 | 110029109 | | G | A | 276 | 15% | MVK | missense_variant | ENST00000228510.3:c.832G>A | ENSP00000228510.3:p.Val278Met |
| YMC50 | 9 | 71098906 | | C | T | 461 | 22% | PGM5 | missense_variant | ENST00000396396.1:c.1421C>T | ENSP00000379678.1:p.Ala474Val |
| YMC50 | 3 | 178927980 | | T | C | 383 | 16% | PIK3CA | missense_variant | ENST00000263967.3:c.1258T>C | ENSP00000263967.3:p.Cys420Arg |
| YMC50 | 3 | 178952018 | | A | G | 409 | 20% | PIK3CA | missense_variant | ENST00000263967.3:c.3073A>G | ENSP00000263967.3:p.Thr1025Ala |
| YMC67 | 5 | 112151261 | | C | T | 238 | 11% | APC | stop_gained | ENST00000257430.4:c.904C>T | ENSP00000257430.4:p.Arg302Ter |
| YMC67 | 5 | 112175951 | | G | GA | 471 | 46% | APC | frameshift_variant | ENST00000257430.4:c.4666dupA | ENSP00000257430.4:p.Thr1556AsnfsTer3 |
| YMC67 | 1 | 207785127 | | A | G | 267 | 52% | CR1 | missense_variant | ENST00000367049.4:c.6401A>G | ENSP00000356016.4:p.Asp2134Gly |
| YMC67 | 17 | 37868208 | | C | T | 168 | 39% | ERBB2 | missense_variant | ENST00000269571.5:c.929C>T | ENSP00000269571.4:p.Ser310Phe |
| YMC67 | 17 | 37879658 | | G | A | 615 | 11% | ERBB2 | missense_variant | ENST00000269571.5:c.2033G>A | ENSP00000269571.4:p.Arg678Gln |
| YMC67 | 6 | 31324602 | | T | A | 226 | 11% | HLA-B | missense_variant | ENST00000412585.2:c.206A>T | ENSP00000399168.2:p.Glu69Val |
| YMC67 | 1 | 11313966 | | C | T | 920 | 22% | MTOR | missense_variant | ENST00000361445.4:c.770G>A | ENSP00000354558.4:p.Arg257Gln |
| YMC67 | 17 | 7577539 | | G | A | 173 | 10% | TP53 | missense_variant | ENST00000269305.4:c.742C>T | ENSP00000269305.4:p.Arg248Trp |
| YMC47 | 1 | 27100181 | | CGCA | C | 363 | 6% | ARID1A | In_Frame_variant | ENST00000324856.7:c.3999_4001del GCA | ENSP00000320485.7:p.Gln1334del |
| YMC45 | 5 | 112175675 | | AAGAG | A | 292 | 51% | APC | frameshift_variant | ENST00000257430.4:c.4391_4394delAGAG | ENSP00000257430.4:p.Glu1464ValfsTer8 |
| YMC45 | 17 | 7578386 | | AGCGCTCATGGTGGGG | A | 192 | 49% | TP53 | In_Frame_variant | ENST00000269305.4:c.529_543delCCCCACCATGAGCGC | ENSP00000269305.4:p.Pro177_Arg181del |
| YMC43 | 1 | 27100181 | | CGCA | C | 133 | 5% | ARID1A | In_Frame_variant | ENST00000324856.7:c.3999_4001del GCA | ENSP00000320485.7:p.Gln1334del |
| YMC42 | 3 | 49405947 | | T | C | 336 | 8% | RHOA | missense_variant | ENST00000418115.1:c.191A>G | ENSP00000400175.1:p.Glu64Gly |
| YMC42 | 17 | 7578413 | | C | A | 289 | 8% | TP53 | missense_variant | ENST00000269305.4:c.517G>T | ENSP00000269305.4:p.Val173Leu |
| YMC42 | 17 | 7579414 | | C | T | 129 | 6% | TP53 | stop_gained | ENST00000269305.4:c.273G>A | ENSP00000269305.4:p.Trp91Ter |
| YMC66 | 16 | 68771347 | | CGCT | C | 78 | 6% | CDH1 | In_Frame_variant | ENST00000261769.5:c.44_46delTGC | ENSP00000261769.4:p.Leu15del |
| YMC66 | 17 | 7579437 | | C | CA | 208 | 30% | TP53 | frameshift_variant | ENST00000269305.4:c.249_250insT | ENSP00000269305.4:p.Ala84CysfsTer65 |
| YMC41 | X | 39921408 | | G | A | 75 | 5% | BCOR | missense_variant | ENST00000342274.4:c.4310C>T | ENSP00000345923.4:p.Ala1437Val |
| YMC41 | 16 | 68771347 | | CGCT | C | 72 | 6% | CDH1 | In_Frame_variant | ENST00000261769.5:c.44_46delTGC | ENSP00000261769.4:p.Leu15del |
| YMC41 | 17 | 7577106 | | G | A | 480 | 68% | TP53 | missense_variant | ENST00000269305.4:c.832C>T | ENSP00000269305.4:p.Pro278Ser |
| YMC40 | 5 | 112175444 | | A | G | 686 | 39% | APC | missense_variant | ENST00000257430.4:c.4153A>G | ENSP00000257430.4:p.Ser1385Gly |
| YMC40 | 5 | 112176039 | | T | TG | 575 | 37% | APC | frameshift_variant | ENST00000257430.4:c.4749dupG | ENSP00000257430.4:p.Pro1584AlafsTer7 |
| YMC40 | 17 | 7577114 | | C | T | 659 | 47% | TP53 | missense_variant | ENST00000269305.4:c.824G>A | ENSP00000269305.4:p.Cys275Tyr |
| YMC39 | 1 | 27100181 | | CGCA | C | 306 | 6% | ARID1A | In_Frame_variant | ENST00000324856.7:c.3999_4001del GCA | ENSP00000320485.7:p.Gln1334del |
| YMC38 | 2 | 30143174 | | C | T | 71 | 6% | ALK | missense_variant | ENST00000389048.3:c.352G>A | ENSP00000373700.3:p.Ala118Thr |
| YMC38 | 1 | 27101174 | | C | T | 219 | 29% | ARID1A | stop_gained | ENST00000324856.7:c.4456C>T | ENSP00000320485.7:p.Gln1486Ter |
| YMC38 | 1 | 27100181 | | CGCA | C | 151 | 5% | ARID1A | In_Frame_variant | ENST00000324856.7:c.3999_4001del GCA | ENSP00000320485.7:p.Gln1334del |
| YMC38 | X | 39934178 | | GT | G | 242 | 32% | BCOR | frameshift_variant | ENST00000342274.4:c.420delA | ENSP00000345923.4:p.Lys140AsnfsTer21 |
| YMC38 | 7 | 140549988 | | T | C | 515 | 7% | BRAF | missense_variant | ENST00000288602.6:c.163A>G | ENSP00000288602.6:p.Lys55Glu |
| YMC38 | 1 | 207787753 | | C | T | 164 | 6% | CR1 | stop_gained | ENST00000367049.4:c.6580C>T | ENSP00000356016.4:p.Arg2194Ter |
| YMC38 | 4 | 185340659 | | CT | C | 99 | 17% | IRF2 | frameshift_variant | ENST00000393593.3:c.150delA | ENSP00000377218.3:p.Asp51MetfsTer19 |
| YMC38 | 9 | 5029792 | | C | A | 122 | 25% | JAK2 | missense_variant | ENST00000381652.3:c.236C>A | ENSP00000371067.3:p.Pro79His |
| YMC38 | 4 | 55968082 | | C | G | 226 | 31% | KDR | missense_variant | ENST00000263923.4:c.2248G>C | ENSP00000263923.4:p.Ala750Pro |
| YMC38 | 12 | 25398281 | | C | T | 264 | 28% | KRAS | missense_variant | ENST00000256078.4:c.38G>A | ENSP00000256078.4:p.Gly13Asp |
| YMC38 | 1 | 11188147 | | T | C | 387 | 16% | MTOR | missense_variant | ENST00000361445.4:c.5947A>G | ENSP00000354558.4:p.Thr1983Ala |
| YMC38 | 9 | 70993145 | | A | G | 371 | 6% | PGM5 | missense_variant | ENST00000396392.1:c.292A>G | ENSP00000379674.1:p.Ile98Val |
| YMC38 | 3 | 178947116 | | T | C | 615 | 42% | PIK3CA | missense_variant | ENST00000263967.3:c.2552T>C | ENSP00000263967.3:p.Val851Ala |
| YMC38 | 10 | 89720811 | | CA | C | 142 | 49% | PTEN | frameshift_variant | ENST00000371953.3:c.968delA | ENSP00000361021.3:p.Asn323MetfsTer21 |
| YMC37 | 4 | 55961110 | | G | A | 788 | 9% | KDR | stop_gained | ENST00000263923.4:c.2830C>T | ENSP00000263923.4:p.Arg944Ter |
| YMC35 | 5 | 112170796 | | T | C | 455 | 22% | APC | missense_variant | ENST00000257430.4:c.1892T>C | ENSP00000257430.4:p.Ile631Thr |
| YMC35 | 18 | 48604707 | | G | A | 218 | 31% | SMAD4 | missense_variant | ENST00000342988.3:c.1529G>A | ENSP00000341551.3:p.Gly510Glu |
| YMC35 | 17 | 7578554 | | A | G | 154 | 30% | TP53 | missense_variant | ENST00000269305.4:c.376T>C | ENSP00000269305.4:p.Tyr126His |
| YMC34 | 17 | 7578190 | | T | C | 827 | 28% | TP53 | missense_variant | ENST00000269305.4:c.659A>G | ENSP00000269305.4:p.Tyr220Cys |
| YMC33 | 16 | 68845617 | | A | T | 938 | 20% | CDH1 | missense_variant | ENST00000261769.5:c.863A>T | ENSP00000261769.4:p.Asp288Val |
| YMC33 | 4 | 153249384 | | C | T | 674 | 13% | FBXW7 | missense_variant | ENST00000263981.5:c.1154G>A | ENSP00000263981.4:p.Arg385His |
| YMC33 | 10 | 89711875 | | G | A | 428 | 13% | PTEN | missense_variant | ENST00000371953.3:c.493G>A | ENSP00000361021.3:p.Gly165Arg |
| YMC33 | 17 | 7577557 | | A | G | 245 | 16% | TP53 | missense_variant | ENST00000269305.4:c.724T>C | ENSP00000269305.4:p.Cys242Arg |
| YMC32 | 5 | 112177688 | | G | A | 471 | 15% | APC | missense_variant | ENST00000257430.4:c.6397G>A | ENSP00000257430.4:p.Asp2133Asn |
| YMC32 | 1 | 27100181 | | CGCA | C | 373 | 6% | ARID1A | In_Frame_variant | ENST00000324856.7:c.3999_4001del GCA | ENSP00000320485.7:p.Gln1334del |
| YMC32 | X | 39922966 | | G | A | 437 | 29% | BCOR | stop_gained | ENST00000342274.4:c.3640C>T | ENSP00000345923.4:p.Gln1214Ter |
| YMC32 | 3 | 178921548 | | G | A | 335 | 16% | PIK3CA | missense_variant | ENST00000263967.3:c.1030G>A | ENSP00000263967.3:p.Val344Met |
| YMC32 | 3 | 178936082 | | G | A | 285 | 14% | PIK3CA | missense_variant | ENST00000263967.3:c.1624G>A | ENSP00000263967.3:p.Glu542Lys |
| YMC32 | 3 | 49413009 | | C | T | 418 | 18% | RHOA | missense_variant | ENST00000418115.1:c.14G>A | ENSP00000400175.1:p.Arg5Gln |
| YMC31 | 17 | 7577035 | | TG | T | 804 | 40% | TP53 | frameshift_variant | ENST00000269305.4:c.902delC | ENSP00000269305.4:p.Pro301GlnfsTer44 |
| YMC65 | X | 39931937 | | T | G | 105 | 16% | BCOR | missense_variant | ENST00000342274.4:c.2662A>C | ENSP00000345923.4:p.Asn888His |
| YMC65 | 19 | 42795360 | | G | T | 58 | 5% | CIC | missense_variant | ENST00000160740.3:c.2440G>T | ENSP00000160740.3:p.Gly814Cys |
| YMC66 | 4 | 153249384 | | C | T | 575 | 7% | FBXW7 | missense_variant | ENST00000263981.5:c.1154G>A | ENSP00000263981.4:p.Arg385His |
| YMC67 | 3 | 49412973 | | C | T | 414 | 7% | RHOA | missense_variant | ENST00000418115.1:c.50G>A | ENSP00000400175.1:p.Gly17Glu |
| YMC68 | 17 | 7577120 | | C | T | 458 | 17% | TP53 | missense_variant | ENST00000269305.4:c.818G>A | ENSP00000269305.4:p.Arg273His |
| YMC64 | 5 | 112174597 | | C | A | 171 | 5% | APC | stop_gained | ENST00000257430.4:c.3306C>A | ENSP00000257430.4:p.Tyr1102Ter |
| YMC64 | 12 | 56495327 | | C | T | 55 | 5% | ERBB3 | missense_variant | ENST00000267101.3:c.3517C>T | ENSP00000267101.3:p.Arg1173Trp |
| YMC30 | 1 | 27023484 | | G | C | 179 | 16% | ARID1A | missense_variant | ENST00000324856.7:c.590G>C | ENSP00000320485.7:p.Gly197Ala |
| YMC30 | 1 | 27100181 | | CGCA | C | 268 | 6% | ARID1A | In_Frame_variant | ENST00000324856.7:c.3999_4001del GCA | ENSP00000320485.7:p.Gln1334del |
| YMC30 | 17 | 7574003 | | G | A | 109 | 44% | TP53 | stop_gained | ENST00000269305.4:c.1024C>T | ENSP00000269305.4:p.Arg342Ter |
| YMC29 | 1 | 27092839 | | A | G | 613 | 17% | ARID1A | missense_variant | ENST00000324856.7:c.2860A>G | ENSP00000320485.7:p.Met954Val |
| YMC29 | 1 | 27105675 | | GGAA | G | 169 | 8% | ARID1A | In_Frame_variant | ENST00000324856.7:c.5299_5301delGAA | ENSP00000320485.7:p.Glu1767del |
| YMC29 | X | 39932171 | | G | A | 446 | 51% | BCOR | stop_gained | ENST00000342274.4:c.2428C>T | ENSP00000345923.4:p.Arg810Ter |
| YMC29 | 4 | 185310160 | | T | C | 472 | 23% | IRF2 | missense_variant | ENST00000393593.3:c.802A>G | ENSP00000377218.3:p.Thr268Ala |
| YMC29 | 3 | 178916636 | | G | A | 461 | 18% | PIK3CA | missense_variant | ENST00000263967.3:c.23G>A | ENSP00000263967.3:p.Gly8Asp |
| YMC29 | 3 | 178952085 | | A | G | 731 | 6% | PIK3CA | missense_variant | ENST00000263967.3:c.3140A>G | ENSP00000263967.3:p.His1047Arg |
| YMC29 | 3 | 114058002 | | AG | A | 296 | 17% | ZBTB20 | frameshift_variant | ENST00000357258.3:c.1856delC | ENSP00000349803.3:p.Pro619LeufsTer43 |
| YMC28 | 12 | 52370254 | | C | A | 59 | 5% | ACVR1B | missense_variant | ENST00000257963.4:c.475C>A | ENSP00000257963.4:p.Arg159Ser |
| YMC28 | 4 | 153244291 | | C | A | 79 | 5% | FBXW7 | missense_variant | ENST00000263981.5:c.1626G>T | ENSP00000263981.4:p.Lys542Asn |
| YMC28 | 4 | 153332615 | | T | C | 57 | 5% | FBXW7 | missense_variant | ENST00000281708.4:c.341A>G | ENSP00000281708.3:p.Glu114Gly |
| YMC27 | 17 | 7577580 | | T | C | 230 | 53% | TP53 | missense_variant | ENST00000269305.4:c.701A>G | ENSP00000269305.4:p.Tyr234Cys |
| YMC26 | 1 | 27100181 | | C | CGCA | 963 | 33% | ARID1A | In_Frame_variant | ENST00000324856.7:c.3999_4001dupGCA | ENSP00000320485.7:p.Gln1334dup |
| YMC26 | 16 | 68844172 | | G | C | 1289 | 5% | CDH1 | missense_variant | ENST00000261769.5:c.760G>C | ENSP00000261769.4:p.Asp254His |
| YMC25 | 1 | 27023193 | | TG | T | 58 | 5% | ARID1A | frameshift_variant | ENST00000324856.7:c.300delG | ENSP00000320485.7:p.Lys101ArgfsTer13 |
| YMC25 | 16 | 68771347 | | CGCT | C | 57 | 5% | CDH1 | In_Frame_variant | ENST00000261769.5:c.44_46delTGC | ENSP00000261769.4:p.Leu15del |
| YMC23 | 1 | 27100181 | | CGCA | C | 268 | 7% | ARID1A | In_Frame_variant | ENST00000324856.7:c.3999_4001del GCA | ENSP00000320485.7:p.Gln1334del |
| YMC22 | 2 | 30142964 | | G | A | 248 | 35% | ALK | missense_variant | ENST00000389048.3:c.562C>T | ENSP00000373700.3:p.Arg188Cys |
| YMC22 | 1 | 27088769 | | T | TG | 110 | 39% | ARID1A | frameshift_variant | ENST00000324856.7:c.2382dupG | ENSP00000320485.7:p.Ser795GlufsTer22 |
| YMC22 | 1 | 27100181 | | CGCA | C | 215 | 6% | ARID1A | In_Frame_variant | ENST00000324856.7:c.3999_4001del GCA | ENSP00000320485.7:p.Gln1334del |
| YMC22 | X | 39934068 | | GCT | G | 335 | 38% | BCOR | frameshift_variant | ENST00000342274.4:c.529_530delAG | ENSP00000345923.4:p.Ser177ProfsTer8 |
| YMC22 | 19 | 42796882 | | GC | G | 169 | 37% | CIC | frameshift_variant | ENST00000160740.3:c.3344delC | ENSP00000160740.3:p.Pro1115GlnfsTer44 |
| YMC22 | 1 | 207737245 | | G | A | 157 | 38% | CR1 | missense_variant | ENST00000367049.4:c.3623G>A | ENSP00000356016.4:p.Arg1208His |
| YMC22 | 12 | 56477655 | | C | T | 228 | 43% | ERBB3 | missense_variant | ENST00000267101.3:c.203C>T | ENSP00000267101.3:p.Thr68Met |
| YMC22 | 10 | 123310860 | | G | A | 383 | 42% | FGFR2 | missense_variant | ENST00000336553.6:c.301C>T | ENSP00000337665.6:p.Arg101Trp |
| YMC22 | 2 | 47657068 | | G | A | 235 | 12% | MSH2 | missense_variant | ENST00000233146.2:c.1264G>A | ENSP00000233146.2:p.Glu422Lys |
| YMC22 | 12 | 110019239 | | GC | G | 173 | 43% | MVK | frameshift_variant | ENST00000228510.3:c.417delC | ENSP00000228510.3:p.Ala141ArgfsTer18 |
| YMC22 | 9 | 70993145 | | A | G | 700 | 13% | PGM5 | missense_variant | ENST00000396392.1:c.292A>G | ENSP00000379674.1:p.Ile98Val |
| YMC22 | 3 | 178921567 | | A | G | 247 | 39% | PIK3CA | missense_variant | ENST00000263967.3:c.1049A>G | ENSP00000263967.3:p.Asp350Gly |
| YMC22 | 3 | 178952084 | | C | T | 384 | 40% | PIK3CA | missense_variant | ENST00000263967.3:c.3139C>T | ENSP00000263967.3:p.His1047Tyr |
| YMC22 | 3 | 114058002 | | AG | A | 88 | 22% | ZBTB20 | frameshift_variant | ENST00000357258.3:c.1856delC | ENSP00000349803.3:p.Pro619LeufsTer43 |
| YMC21 | 17 | 37866117 | | C | A | 298 | 17% | ERBB2 | missense_variant | ENST00000269571.5:c.626C>A | ENSP00000269571.4:p.Ser209Tyr |
| YMC20 | 5 | 112173917 | | C | T | 620 | 10% | APC | stop_gained | ENST00000257430.4:c.2626C>T | ENSP00000257430.4:p.Arg876Ter |
| YMC20 | 5 | 112175650 | | T | TA | 394 | 6% | APC | frameshift_variant | ENST00000257430.4:c.4364dupA | ENSP00000257430.4:p.Asn1455LysfsTer2 |
| YMC19 | 1 | 27100181 | | CGCA | C | 160 | 5% | ARID1A | In_Frame_variant | ENST00000324856.7:c.3999_4001del GCA | ENSP00000320485.7:p.Gln1334del |
| YMC19 | 1 | 11300361 | | T | A | 74 | 5% | MTOR | missense_variant | ENST00000361445.4:c.1785A>T | ENSP00000354558.4:p.Glu595Asp |
| YMC19 | 17 | 7577114 | | C | T | 346 | 73% | TP53 | missense_variant | ENST00000269305.4:c.824G>A | ENSP00000269305.4:p.Cys275Tyr |
| YMC18 | 1 | 27100181 | | CGCA | C | 281 | 5% | ARID1A | In_Frame_variant | ENST00000324856.7:c.3999_4001del GCA | ENSP00000320485.7:p.Gln1334del |
| YMC17 | 2 | 29448360 | | C | T | 88 | 8% | ALK | missense_variant | ENST00000389048.3:c.3139G>A | ENSP00000373700.3:p.Ala1047Thr |
| YMC17 | 17 | 7578263 | | G | A | 396 | 56% | TP53 | stop_gained | ENST00000269305.4:c.586C>T | ENSP00000269305.4:p.Arg196Ter |
| YMC16 | 3 | 49412898 | | T | C | 1858 | 11% | RHOA | missense_variant | ENST00000418115.1:c.125A>G | ENSP00000400175.1:p.Tyr42Cys |
| YMC15 | 12 | 52369259 | | G | GC | 290 | 79% | ACVR1B | frameshift_variant | ENST00000257963.4:c.303dupC | ENSP00000257963.4:p.Asn102GlnfsTer16 |
| YMC15 | 5 | 112175675 | | AAGAG | A | 130 | 58% | APC | frameshift_variant | ENST00000257430.4:c.4391_4394delAGAG | ENSP00000257430.4:p.Glu1464ValfsTer8 |
| YMC15 | 1 | 27100181 | | CGCA | C | 207 | 5% | ARID1A | In_Frame_variant | ENST00000324856.7:c.3999_4001del GCA | ENSP00000320485.7:p.Gln1334del |
| YMC15 | 7 | 140482926 | | AG | A | 214 | 18% | BRAF | frameshift_variant | ENST00000288602.6:c.1208delC | ENSP00000288602.6:p.Pro403LeufsTer8 |
| YMC15 | 13 | 31480888 | | G | A | 60 | 7% | MEDAG | missense_variant | ENST00000380482.4:c.236G>A | ENSP00000369849.4:p.Arg79His |
| YMC15 | 13 | 31480851 | | CG | C | 60 | 15% | MEDAG | frameshift_variant | ENST00000380482.4:c.206delG | ENSP00000369849.4:p.Gly69AlafsTer29 |
| YMC15 | 12 | 110019239 | | GC | G | 190 | 26% | MVK | frameshift_variant | ENST00000228510.3:c.417delC | ENSP00000228510.3:p.Ala141ArgfsTer18 |
| YMC15 | 17 | 7579546 | | CG | C | 390 | 81% | TP53 | frameshift_variant | ENST00000269305.4:c.140delC | ENSP00000269305.4:p.Pro47ArgfsTer76 |
| YMC14 | 11 | 69465987 | | AGAG | A | 75 | 5% | CCND1 | In_Frame_variant | ENST00000227507.2:c.839_841delAGG | ENSP00000227507.2:p.Glu280del |
| YMC14 | 1 | 207787753 | | C | T | 601 | 5% | CR1 | stop_gained | ENST00000367049.4:c.6580C>T | ENSP00000356016.4:p.Arg2194Ter |
| YMC14 | 3 | 41268766 | | A | T | 640 | 6% | CTNNB1 | missense_variant | ENST00000349496.5:c.1004A>T | ENSP00000344456.5:p.Lys335Ile |
| YMC14 | 17 | 7574029 | | C | CG | 313 | 14% | TP53 | frameshift_variant | ENST00000269305.4:c.997dupC | ENSP00000269305.4:p.Arg333ProfsTer4 |
| YMC13 | 5 | 112155000 | | AG | A | 520 | 31% | APC | frameshift_variant | ENST00000257430.4:c.1273delG | ENSP00000257430.4:p.Glu425LysfsTer29 |
| YMC13 | 1 | 27100181 | | CGCA | C | 283 | 6% | ARID1A | In_Frame_variant | ENST00000324856.7:c.3999_4001del GCA | ENSP00000320485.7:p.Gln1334del |
| YMC13 | 19 | 42799299 | | TC | T | 66 | 6% | CIC | frameshift_variant | ENST00000160740.3:c.4784delC | ENSP00000160740.3:p.Pro1595HisfsTer23 |
| YMC12 | 6 | 31322976 | | G | C | 444 | 77% | HLA-B | missense_variant | ENST00000412585.2:c.920C>G | ENSP00000399168.2:p.Pro307Arg |
| YMC12 | 9 | 5022119 | | C | A | 755 | 21% | JAK2 | stop_gained | ENST00000381652.3:c.132C>A | ENSP00000371067.3:p.Tyr44Ter |
| YMC12 | 17 | 7578203 | | C | T | 997 | 30% | TP53 | missense_variant | ENST00000269305.4:c.646G>T | ENSP00000269305.4:p.Val216Leu |
| YMC11 | 17 | 7578493 | | C | T | 423 | 7% | TP53 | stop_gained | ENST00000269305.4:c.437G>A | ENSP00000269305.4:p.Trp146Ter |
| YMC10 | 17 | 37879658 | | G | A | 513 | 5% | ERBB2 | missense_variant | ENST00000269571.5:c.2033G>A | ENSP00000269571.4:p.Arg678Gln |
| YMC10 | 12 | 56482341 | | G | T | 220 | 8% | ERBB3 | missense_variant | ENST00000267101.3:c.889G>T | ENSP00000267101.3:p.Asp297Tyr |
| YMC10 | 3 | 49412898 | | T | C | 654 | 16% | RHOA | missense_variant | ENST00000418115.1:c.125A>G | ENSP00000400175.1:p.Tyr42Cys |
| YMC9 | 11 | 69465987 | | AGAG | A | 64 | 6% | CCND1 | In_Frame_variant | ENST00000227507.2:c.839_841delAGG | ENSP00000227507.2:p.Glu280del |
| YMC9 | 17 | 7577094 | | G | A | 776 | 35% | TP53 | missense_variant | ENST00000269305.4:c.844C>T | ENSP00000269305.4:p.Arg282Trp |
| YMC8 | 17 | 7577108 | | C | T | 464 | 6% | TP53 | missense_variant | ENST00000269305.4:c.830G>A | ENSP00000269305.4:p.Cys277Tyr |
| YMC6 | 1 | 27023908 | | GGCTGCGGCGGCGGCA | G | 65 | 12% | ARID1A | In_Frame_variant | ENST00000324856.7:c.1029_1043delAGCTGCGGCGGCGGC | ENSP00000320485.7:p.Ala345_Ala349del |
| YMC5 | 1 | 27100181 | | CGCA | C | 333 | 5% | ARID1A | In_Frame_variant | ENST00000324856.7:c.3999_4001del GCA | ENSP00000320485.7:p.Gln1334del |
| YMC4 | 1 | 27023391 | | C | T | 73 | 5% | ARID1A | missense_variant | ENST00000324856.7:c.497C>T | ENSP00000320485.7:p.Ala166Val |
| YMC4 | 1 | 27100181 | | CGCA | C | 155 | 6% | ARID1A | In_Frame_variant | ENST00000324856.7:c.3999_4001del GCA | ENSP00000320485.7:p.Gln1334del |
| YMC4 | 3 | 178952085 | | A | G | 679 | 48% | PIK3CA | missense_variant | ENST00000263967.3:c.3140A>G | ENSP00000263967.3:p.His1047Arg |
| YMC4 | 3 | 49413010 | | G | A | 228 | 9% | RHOA | missense_variant | ENST00000418115.1:c.13C>T | ENSP00000400175.1:p.Arg5Trp |
| YMC3 | 19 | 42799177 | | C | A | 57 | 5% | CIC | missense_variant | ENST00000160740.3:c.4655C>A | ENSP00000160740.3:p.Ala1552Glu |
| YMC2 | 1 | 27100181 | | CGCA | C | 246 | 5% | ARID1A | In_Frame_variant | ENST00000324856.7:c.3999_4001del GCA | ENSP00000320485.7:p.Gln1334del |
| YMC1 | 1 | 207787753 | | C | T | 489 | 8% | CR1 | stop_gained | ENST00000367049.4:c.6580C>T | ENSP00000356016.4:p.Arg2194Ter |
| YMC1 | 12 | 25398281 | | C | T | 1253 | 46% | KRAS | missense_variant | ENST00000256078.4:c.38G>A | ENSP00000256078.4:p.Gly13Asp |
| YMC1 | 17 | 7577124 | | C | A | 440 | 36% | TP53 | missense_variant | ENST00000269305.4:c.814G>T | ENSP00000269305.4:p.Val272Leu |
